# Revealing the association between vitamin D metabolic pathway gene variants and lung cancer risk: a systematic review and meta-analysis

**DOI:** 10.3389/fgene.2024.1302527

**Published:** 2024-02-28

**Authors:** Mohamed I. Elsalahaty, Samar Sami Alkafaas, Aya O. Bashir, Khaled A. El-Tarabily, Mohamed T. El-Saadony, Eman H. Yousef

**Affiliations:** ^1^ Biochemistry Division, Department of Chemistry, Faculty of Science, Tanta University, Tanta, Egypt; ^2^ Molecular Cell Biology Unit, Division of Biochemistry, Chemistry Department, Faculty of Science, Tanta University, Tanta, Egypt; ^3^ Department of Biochemistry, Faculty of Pharmacy, Mansoura University, Mansoura, Egypt; ^4^ Department of Biology, College of Science, United Arab Emirates University, Al Ain, United Arab Emirates; ^5^ Department of Agricultural Microbiology, Faculty of Agriculture, Zagazig University, Zagazig, Egypt; ^6^ Department of Biochemistry, Faculty of Pharmacy, Horus University-Egypt, Damietta, Egypt

**Keywords:** gene variants, lung cancer, polymorphism, vitamin D, vitamin D receptor

## Abstract

Lung cancer is a crucial global issue, with more than one million deaths annually. While smoking is considered the main etiology of the disease, several genetic variants are associated with it. Alterations in vitamin D pathway genes have also been studied in regards to lung cancer, but the findings have been inconclusive. We here present a systematic review and meta-analysis of seven genes in this pathway: *CYP2R1*, *CYP27B1*, *CYP24A1*, *CYP3A4*, *CYP3A5*, *GC*, and *VDR*. Four databases (PubMed, Scopus, Cochrane Library, and Web of Science (WOS) databases) were searched. From these, 16 eligible case–control studies comprising 6,206 lung cancer cases and 7,272 health controls were obtained. These studies were subjected to comprehensive data extraction and quality scoring, and the pooled odds ratio with a 95% confidence interval was calculated to estimate the effect of each variant along with heterogeneity analysis and a risk of bias assessment. Our meta-analysis revealed an association between *CYP3A4* (rs2740574) and lung cancer in the allelic, heterozygous, and dominant models. In addition, both *VDR* (Fok1: rs2228570) and *VDR* (Cdx-2: rs11568820) displayed a protective role in lung cancer development in the heterozygous and dominant models. Furthermore, *VDR* (Taq1: rs731236) showed a decreased risk of lung cancer in the allelic, homozygous, and recessive models. Similarly, *VDR* (BsmI: rs1544410) had a positive effect on lung cancer risk when subjected to allelic and recessive models. Our meta-analysis revealed the lack of association of *CYP2R1* (rs10741657), *CYP27B1* (rs3782130), *CYP27B1* (rs10877012), *CYP24A1* (rs6068816), *CYP24A1* (rs4809960), *CYP3A5* (rs776746), *GC* (rs7041), *GC* (rs4588), and *VDR* (ApaI: rs7975232) with lung cancer. Our work revealed that *CYP3A4* (rs2740574) can represent an independent risk factor for lung cancer. This conclusion can aid better personalized medicine for lung cancer management, while further assessment for genetic variants of *CYP3A4*, *CYP27B1*, *CYP24A1*, *GC*, and *VDR* is still required to address more robust evidence.

## 1 Introduction

Lung cancer is the most robust lethal carcinoma worldwide, accounting for 1.76 million deaths annually (Bade and Dela Cruz, 2020). Small-cell lung cancer (SCLC) and non-small-cell lung cancer (NSCLC) are two primary histological subtypes of lung cancer. NSCLC accounts for 80%–85% of lung cancer cases and includes adenocarcinoma, squamous cell carcinoma, and large-cell carcinoma (Ben et al., 2021). Smoking has been identified as the leading factor contributing to the incidence of lung cancer. Other risk factors include viral infections, exposure to heavy metals, radiation, asbestos, and air pollution (Li et al., 2019). However, these etiologies rarely account for lung cancer, suggesting that genetic factors play a significant role in its incidence (Zhu et al., 2021). Genome-wide association studies (GWASs) in populations from Europe and Asia have conclusively proven the relationship between genetic polymorphisms and the risk of developing lung cancer (Zhou et al., 2020). Therefore, the identification of vulnerable genes and high-risk populations is one of the main goals of lung cancer research for achieving early prevention and treatment (Wei et al., 2021).

**FIGURE 1:**
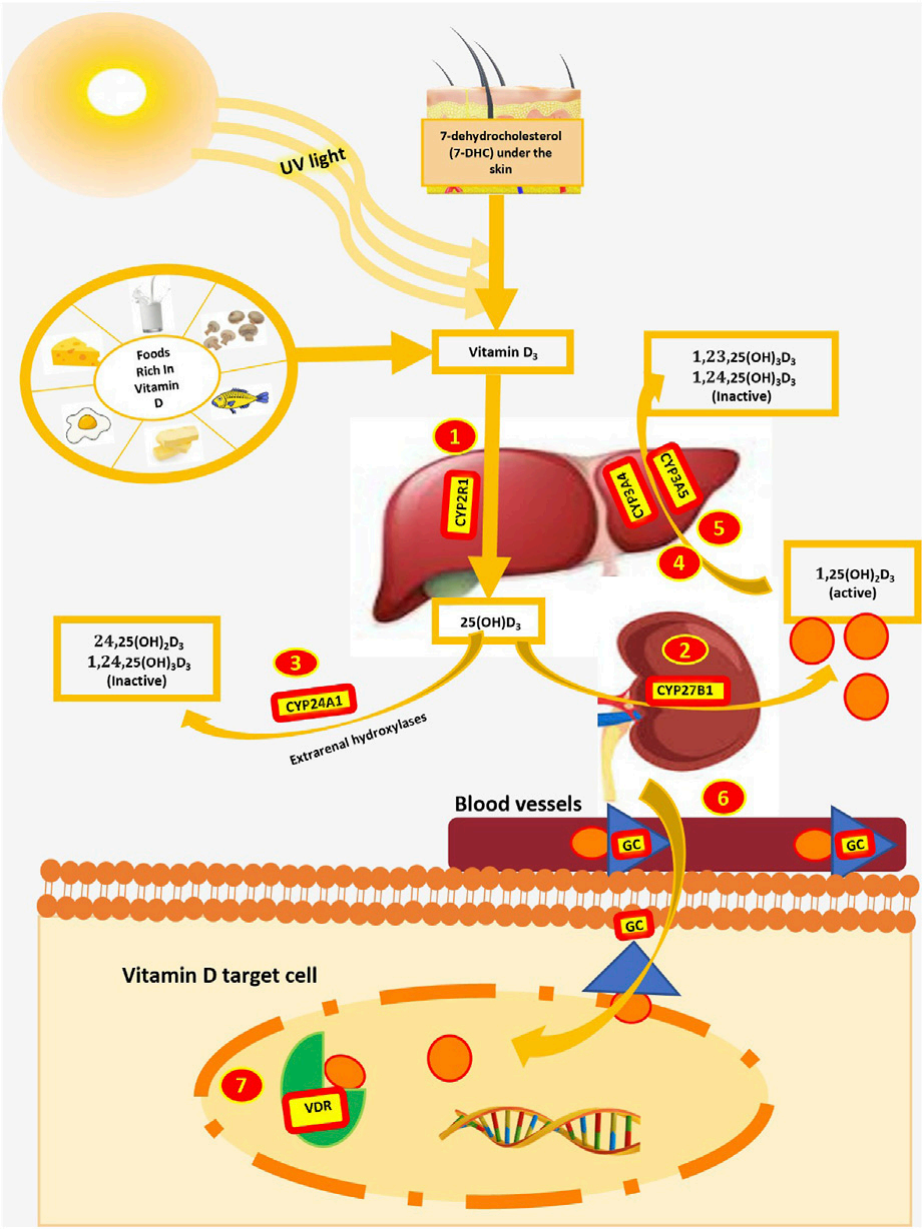
Genes involved in the synthesis and metabolism of vitamin D. UV light, ultraviolet light; *GC*, group-specific component; *VDR*, vitamin D receptor.

Vitamin D is a seco-steroidal prohormone which is synthesized and metabolized by a series of reactions catalyzed by several enzymes. First, pro-vitamin D, absorbed from food or produced in the skin after exposure to sunlight, is converted by the vitamin D 25-hydroxylase enzyme *CYP2R1* in the liver (Janoušek et al., 2022). A summary of this process is shown in Figure 1. The 25(OH) D produced is then converted into 1,25-dihydroxyvitamin D [1.25(OH)_2_D_3_] by the 25-hydroxyvitamin D-1α-hydroxylase enzyme that is encoded by the *CYP27B1* gene in the kidney. Additionally, 25(OH)D_3_ can also be converted into 24.25(OH)_2_D_3_ by hydroxylation at C-24 through the mitochondrial inner membrane enzyme 1,25-dihydroxyvitamin D (3) 24-hydroxylase encoded by the *CYP24A1* gene (Christakos et al., 2015).

Cytochrome P450 3A4 is a microsomal enzyme encoded by the *CYP3A4* gene that plays a plethora of roles in the xenobiotic transformation process of many drugs and endogenous substances. It is involved in the conversion of vitamin D metabolites into their corresponding inactive molecules, such as 4β,25-OH-D3. Additionally, it activates 23R- and 24S-mediated conversion of 1α,25(OH)2D3 into inactive 1α,23R,25(OH)_2_D_3_ and 1α,24S,25(OH)_2_D_3_ (Jones et al., 2014). Thus, *CYP3A4* was extensively studied in the metabolism of vitamin D (Wang et al., 2012; Piotrowska et al., 2019; Kasarla et al., 2022a). Nevertheless, vitamin D inactivation is mainly dependent on *CYP3A4*, and the cytochrome P450 3A5 enzyme encoded by the *CYP3A5* gene catalyzes 23- or 24-hydroxylation of 1,25-(OH)_2_D_3_ (Xu et al., 2006; Klyushova et al., 2022).

Additionally, 1,25-dihydroxyvitamin D (3) 24-hydroxylase encoded by the *CYP24A1* gene is considered a factor in the vitamin D transformation process and participates in the degradation process of both 25-OH-D3 and 1,25-(OH)_2_D_3_ into 24-hydroxylated products in a tissue-dependent manner (Jones et al., 2012; Wang et al., 2013). In fact, it has been suggested that alterations in the vitamin D metabolism result mainly from the induction of hepatic P450 enzymes, including *CYP3A4* and *CYP24A1* (Wang et al., 2013). Furthermore, in the case of vitamin D toxicity, the liver microsomal enzymes *CYP3A4* and *CYP3A5* and the extrarenal enzyme *CYP24A1* promote the hydroxylation of vitamin D3 into inactive metabolites (Klyushova et al., 2022; Kasarla et al., 2022b). Vitamin D-binding proteins encoded by the *GC* gene transport the active metabolite 1.25- (OH)2D3 to target tissues where it can bind to the vitamin D receptor gene *VDR* and regulate physiological genes (Kasarla et al., 2022b).

Vitamin D is involved in a variety of cellular processes such as proliferation, differentiation, metastasis, angiogenesis, and apoptosis (Wei et al., 2018). Vitamin D levels have been linked to the risk of developing several cancers, including breast (Kim and Je, 2014), colorectal (Garland and Gorham, 2017), and prostate (Gilbert et al., 2011). Additionally, a previous meta-analysis has reported an inverse association between serum vitamin D levels and lung cancer risk (Zhang et al., 2015a). Furthermore, it has been found that vitamin D could suppress the metastatic growth of lung cancer cells in animal models (Nakagawa et al., 2005; Zhang et al., 2015b). Therefore, results from previous reports have revealed the potentially preventive role of vitamin D against lung cancer.

Genetic variations in vitamin D genes are potential modulators of their enzymatic functions, levels of expression, and subsequent roles in the susceptibility, progression, and prognosis of lung cancer (Pineda Lancheros et al., 2021; Pineda Lancheros et al., 2022). *CYP2R1* gene polymorphisms are linked to an increased risk of NSCLC mortality, particularly in elderly NSCLC patients who have not received treatment. Furthermore, the prognosis of NSCLC may be impacted by several genetic variants connected to the Vitamin D pathway (Kong et al., 2020). Furthermore, the activity of *CYP27B1* has been found to be attenuated among some small cell and non-small cell cancer cell lines (Hansdottir et al., 2008). *CYP3A4* and *CYP3A5* activities as part of liver machinery P450 protein content in the liver account for the metabolism of more than 50% of all drugs and exogenous carcinogens (Ingelman-Sundberg et al., 2007). *CYP3A4* and *CYP3A5* affect the activation of benzo [a]pyrene (B [a]P), N9-nitrosonornicotine (NNN), aflatoxin B1, stergmatocystin, alpha-hydroxytamoxifen, and procarcinogens present in tobacco smoke (Islam et al., 2014). Altered levels of these toxins are thought to be involved in lung cancer pathogenesis. Increased *VDR* expression in lung cancer has been correlated with improved survival (Salama et al., 2022). Genetic variants within the *VDR* gene might potentially influence the binding of 1,25(OH)_2_D and subsequently alter vitamin D levels and lung cancer pathogenesis (Haznadar et al., 2018). Nevertheless, despite research into the association between vitamin D gene pathway variants and lung cancer, much is still unknown due to a lack of conclusive findings. So this work extensively investigated the genetic variations in the vitamin D pathway, specifically the polymorphisms of *CYP2R1*, *CYP27B1*, *CYP24A1*, *CYP3A4*, *CYP3A5*, *GC*, and *VDR* genes, to determine their connection with increased risk of developing lung cancer.

## 2 Materials and methods

### 2.1 Search strategy

We conducted a systematic review and meta-analysis in accordance with meta-analysis of observational studies in epidemiology (MOOSE) guidelines (Stroup et al., 2000). The study results were reported following Preferred Reporting Items for Systematic Reviews and Meta-Analyses protocols (PRISMA-2020) (Page et al., 2021). A comprehensive literature search of PubMed, Scopus, the Cochrane Library, and Web of Science (WOS) databases to 1 January 2023 was conducted by two authors independently for all relevant articles on the effects of the *CYP2R1*, *CYP27B1*, *CYP24A1*, *CYP3A4*, *CYP3A5*, *GC*, and *VDR* polymorphisms on lung cancer risk using the following search strategy: (“lung” or “pulmonary”) and (“cancer,” “tumor,” “neoplasm,” or “carcinoma”) and (“polymorphism,” “polymorphic,” “variation,” “variant,” “mutant,” “mutation,” “SNP,” “genotypic,” “genotype,” “allelic,” or “allele”) and (“CYP24A1,” “cytochrome P450 family 24 subfamily A member 1,” “P450-CC24,” “CYP24,” “CP24,” “23-OHase,” “rs6068816,” “rs4809957,” “vitamin D-binding protein,” “vitamin D binding protein,” “Gc-MAF,” “GC,” “DBP,” “VDBP,” “rs7041,” “rs4588,” “CYP2R1,” “25-hydroxylase,” “cytochrome P450 2R1,” “CYP27B1,” “25 hydroxyvitamin D3 1 alpha hydroxylase,” “25-hydroxycholecalciferol 1-hydroxylase,” “calcidiol 1 monooxygenase,” “calcidiol 1-monooxygenase,” “25-hydroxyvitamin D 1-alpha-hydroxylase,” “25 hydroxyvitamin D 1 alpha hydroxylase,” “25-hydroxyvitamin D2 1-hydroxylase,” “1-hydroxylase, 25-hydroxyvitamin D2,” “25 hydroxyvitamin D2 1 hydroxylase,” “cytochrome P-450 CYP27B1,” “cytochrome P 450 CYP27B1,” “25-hydroxycholecalciferol-1-hydroxylase,” “25 hydroxyvitamin D3-1-alpha hydroxylase,” “25-hydroxyvitamin D3 1 alpha-hydroxylase,” “EC 1.14.15.18,” “CYP3A5,” “cytochrome P450, family 3, subfamily A, polypeptide 5,” “cytochrome P450-PCN3,” “cytochrome P450 3A5,” “CYP3A4,” “cytochrome P450 family 3 subfamily A member 4,” “CYP3A3” “cytochrome P450, family 3, subfamily A, polypeptide 4,” “cholesterol 25-hydroxylase,” “albendazole sulfoxidase,” “cytochrome P450 3A4,” “cytochrome P450 3A3,” “nifedipine oxidase,” “EC 1.14.14.1,” “CYPIIIA4,” “taurochenodeoxycholate 6-alpha-hydroxylase,” “glucocorticoid-inducible P450,” “EC 1.14.14.55,” “P450PCN1,” “CYP3A,” “NF-25,” “VDDR3,” “CP33,” “CP34,” “HLP,” “vitamin D receptor,” “vitamin D3 receptor,” “calcitriol receptor,” “1,25-dihydroxyvitamin D3 receptor,” “VDR,” “FokI,” “BsmI,” “ApaI,” or “TaqI”). References cited in recruited articles were manually searched for additional eligible studies. When multiple publications reported the same or overlapping data, we only recruited the publication with complete data or the largest population.

### 2.2 Inclusion and exclusion criteria

The following inclusion criteria were used to identify the eligible studies for our meta-analysis: 1) case–control studies evaluated the potential association between the *CYP2R1*, *CYP27B1*, *CYP24A1*, *CYP3A4*, *CYP3A5*, *GC*, or *VDR* polymorphisms and lung risk; 2) studies with sufficient data to calculate an odds ratio (OR) with 95% confidence interval (95% CI); 3) original full-text articles. Otherwise, studies were excluded if they were 1) review articles, meta-analyses, letters, case reports, or articles with abstract only; 2) articles without controls; 3) non-human studies; 4) had duplicated or republished data.

### 2.3 Quality assessment and data extraction

Two authors independently reviewed and extracted the following baseline information for all included studies: first author’s name, year of publication, country of origin, geographical distribution, source of control [hospital-based (HB) or population-based (PB)], mean/median age of cases and controls, sample size of cases and control, classification of lung cancer, genotyping method, allelic/genotype frequencies of cases and controls, matching criteria, and quality control of genotyping. Potential conflicts were resolved by discussion with two other authors to reach a final consensus. Each study was given quality scores based on certain criteria (Supplementary Table S1) (Xu et al., 2015).

### 2.4 Statistical analysis

First, the allelic and genotypic frequencies of eligible studies were extracted and calculated from the selected records. HWE within cancer-free controls was calculated by the chi-squared method with a *p*-value >0.05 representing equilibrium between subjects (Elsalahaty et al., 2023). Next, the crude odds ratios (ORs) and their 95% confidence intervals (CI) were processed to assess the association of included variants with the risk of lung cancer. Different genetic association models were performed, including the allelic, homozygous, heterozygous, dominant, and recessive models (Elshazli et al., 2018). Heterogeneity testing amongst eligible reports was performed using the Q-statistic test along with the I^2^ index, with a *p*-value for the Q-test ≤0.10 and I^2^ index >50%, which signified strong heterogeneity between studies; hence, the random-effects model was chosen (Higgins et al., 2003; El Awady et al., 2022). Publication bias was evaluated using Egger’s regression method and Begg’s funnel plot to ensure a symmetric fashion; the *p*-value <0.05 was considered significant. We conducted a sensitivity analysis by excluding any included case–control study at a time and then recalculated the significance of the results to detect whether our outcomes were significantly impacted by the presence of each individual study. The present meta-analysis was performed using Comprehensive Meta-analysis version 3.0 (Brüggemann and Rajguru, 2022).

## 3 Results

### 3.1 Characteristics of eligible studies

**FIGURE 2:**
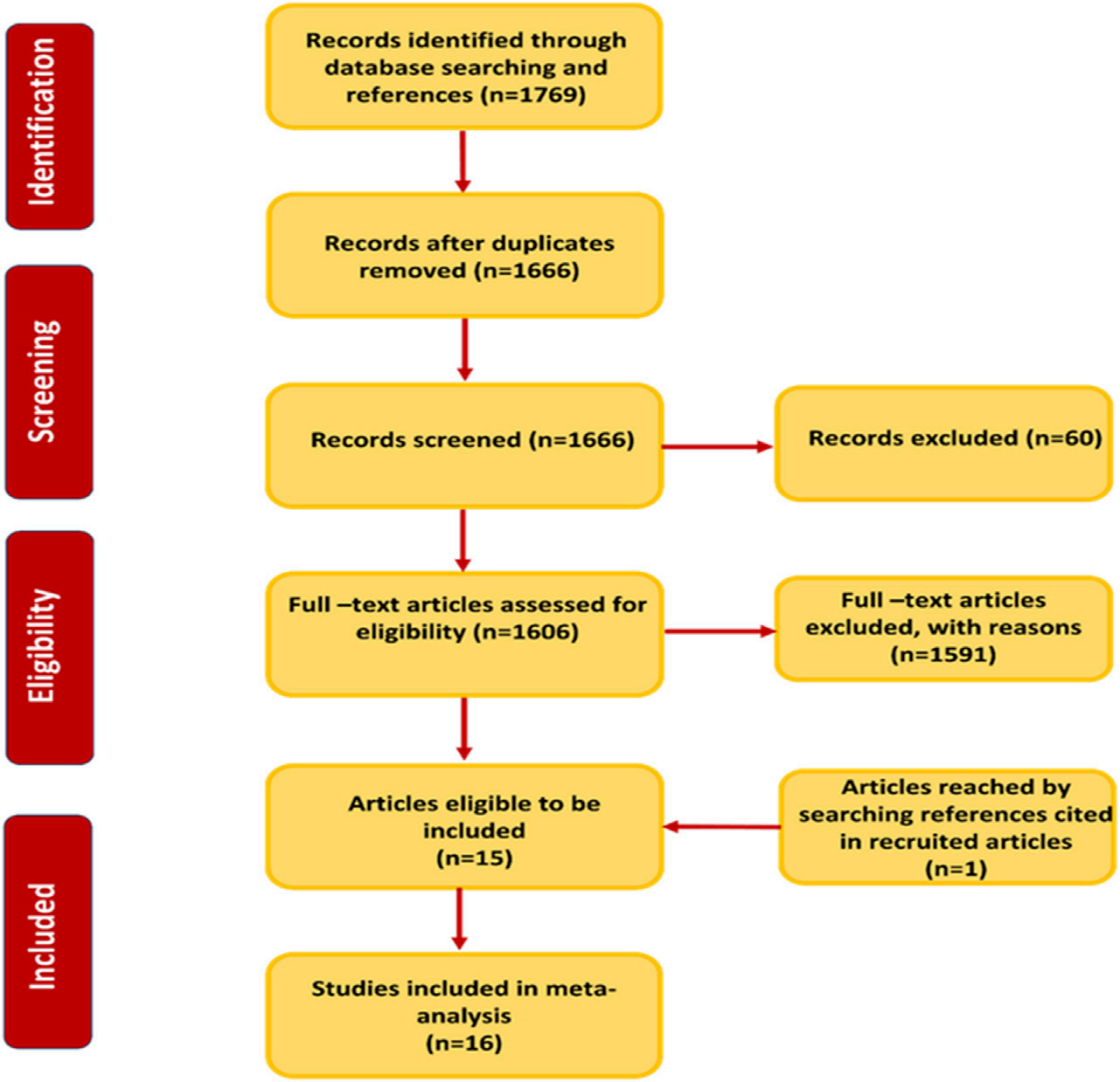
Preferred Reporting Items for Systematic Reviews and Meta-Analyses protocols (PRISMA) flow diagram of the search strategy.

Depending on the keyword-based searching on databases, we acquired 1,769 records from the electronic databases (Figure 2). We removed 103 records due to duplication. The remaining 1,666 were subjected to title and abstract screening, thus excluding 60 records. The 1,606 records were independently screened by two authors, leading to 15 being selected for their eligibility as well as one record obtained from the references of included articles (Figure 2). The research in the 16 articles was performed in ten countries: China (Kong et al., 2015; Wu et al., 2016; Qu et al., 2019; Xiong et al., 2020; Jia et al., 2020), Germany (Dally et al., 2004; Timofeeva et al., 2009), Turkey (Dogan et al., 2009; Çiçek et al., 2017), Norway (Zienolddiny et al., 2008), Bangladesh (Islam et al., 2014), Tunisia (Kaabachi et al., 2014), Thailand (Maneechay et al., 2015), Poland (Gromowski et al., 2017), United States of America (Haznadar et al., 2018), and Spain (Pineda Lancheros et al., 2022). The 16 case–control studies included 6,206 lung cancer cases along with 7,272 healthy controls. The main characteristics of the included studies are presented in Table 1. Additionally, comprehensive data extraction of genotypic and allelic frequencies, along with quality scores for the 16 studies, is summarized in Supplementary Table S2.

**TABLE 1:** Main characteristics of the 16 records included in this meta-analysis.

First author	Year	Country	Studied Genes*variants	Geographical distribution		Ct. source (HB or PB)^a^	Mean age		Sample size		Classification of disease	Genotyping method
							Cases	Ct	Cases	Ct		
Heike Dally	2003	Germany	*CYP3A4* (rs2740574) *CYP3A5* (rs776746)	Europe		HB	NA^b^	NA	782	428	Mixed^c^	Capillary PCR/fluorescence
Shanbeh Zienolddiny	2008	Norway	*CYP3A4* (rs2740574)	Europe		PB	65 (31–85)	60 (50–83)	250	297	NSCLC^d^	Arrayed primer extension
Maria N.Timofeeva	2009	Germany	*CYP3A4* (rs2740574) *CYP3A5* (rs776746)	Europe		PB	45.1 ± 4.3	45.0 ± 4.4	638	1,300	Mixed	Fluorescence-based melting curve analysis
Dogan	2009	Turkey	*VDR* (ApaI: rs7975232; Taq1: rs731236; BsmI: rs1544410)	Europe		HB	NA	NA	137	156	Mixed	PCR-RFLP^e^
Mohammad Safiqul Islam	2013	Bangladesh	*CYP3A4* (rs2740574) *CYP3A5* (rs776746)	Asia		NA	57.87 ± 10.12	58.14 ± 9.77	106	116	Mixed	PCR-RFLP
Kaabachi	2014	Tunisia	*VDR* (ApaI: rs7975232; Fok1: rs2228570; Taq1: rs731236; BsmI: rs1544410)	Africa		NA	58.51 ± 10.25	52.64 ± 6.36	240	280	Mixed	PCR-RFLP
Jinyu Kong	2015	China	*CYP2R1* (rs10741657) *CYP27B1* (rs3782130) *CYP24A1* (rs6068816) *CYP24A1* (rs4809957) *GC* (rs7041) *VDR* (rs11574129)	Asia		HB	60 (23–83)	60 (23–83)	603	661	NSCLC	TaqMan
Wanwisa Maneechay	2015	Thailand	*GC* (rs7041; rs4588)	Asia		PB	62.8 ± 11.7	62.5 ± 11.5	113	113	Mixed	TaqMan
Xiayu Wu	2016	China	*CYP27B1* (rs3782130) *CYP24A1* (rs4809960) *GC* (rs7041; rs4588) *VDR* (ApaI: rs7975232; Fok1: rs2228570; Cdx-2: rs11568820; Taq1: rs731236; BsmI: rs1544410)	Asia		HB	57.4 ± 5.8	59.6 ± 4.7	426	445	NSCLC	PCR-RFLP
Gromowski	2017	Poland	*VDR* (ApaI: rs7975232; Fok1: rs2228570; Cdx-2: rs11568820; Taq1: rs731236; BsmI: rs1544410)	Europe		PB	61 (28–88)	61 (28–88)	840	920	Mixed	TaqMan
Hülya Kanbur	2017	Turkey	*VDR* (Fok1: rs2228570; BsmI: rs1544410)	Europe		NA	60.41 ± 11.42	55.71 ± 8.60	59	55	Mixed	TaqMan
Ruoyi Qu	2018	China	*CYP24A1* (rs6068816)	Asia		HB	56.87 ± 10.31	57.96 ± 10.65	345	351	Mixed	TaqMan
Majda Haznadar	2018	United States	*CYP24A1* (rs2585439; rs3787555; rs3787557; rs2769237; rs6022993; rs8120563; rs10623012; rs2762940; rs2762933; rs2209314)	North America		PB	66.3 ± 10.0	67.0 ± 8.9	406	437	NSCLC	Allele-specific PCR
Zhuoqi Jia	2020	China	*CYP3A4* (rs3735451; rs4646440; rs35564277; rs4646437)	Asia		HB	60.75 ± 9.98	60.40 ± 7.39	507	505	NSCLC	Mass spectrometry
Qiantao Xiong	2020	China	*CYP24A1* (rs6068816; rs4809960; rs2585428; rs6022999)	Asia		HB	58.13 ± 10.25	56.16 ± 10.85	550	800	Mixed	Sanger sequencing
Laura Elena	2022	Spain	*CYP2R1* (rs10741657; rs3782130) *CYP24A1* (rs6068816; rs4809957) *GC* (rs7041) *VDR* (ApaI: rs7975232; Fok1: rs2228570; Cdx-2: rs11568820; Taq1: rs731236; BsmI: rs1544410)	Europe		HB	61.1 ± 10.7	64 (52–75)	204	408	NSCLC	TaqMan

^a^ ^−ΔΔCT^. Source (HB or PB). Control subjects’ source, where HB indicates hospital-based control and PB indicates population-based control.

^b^ NA: not applicable.

^c^ Mixed: unidentified sub-classification of lung cancer.

^d^ NSCLC: non-small-cell lung cancer.

^e^ PCR-RFLP: polymerase chain reaction–restriction fragment length polymorphism.

### 3.2 Association of *CYP2R1*, *CYP27B1*, *CYP24A1*, *CYP3A4*, *CYP3A5*, *GC*, and *VDR* with lung cancer polymorphisms in lung cancer risk

All eligible variants for meta-analysis (≥2 records) were subjected to a five-heredity-model-pooled meta-analysis (Duan et al., 2020) to assess their association with lung cancer. Significant gene variants are depicted in Table 2. Our work addressed statistical significance regarding *CYP24A1* (rs4809957) with risk of lung cancer under a homozygous model [AA *versus* GG, OR = 1.788, 95% CI = 1.172–2.727, *p*-value = 0.007] (Table 2; Supplementary Figure S1A **)**. However, it revealed a protective impact under the heterozygous model [OR = 0.751, 95% CI = 0.599–0.942 [*p*-value = 0.013] (Table 2, Supplementary Figure S1B).

**TABLE 2:** Meta-analysis of the association of *CYP24A1* (rs4809957), *CYP3A4* (rs2740574), *VDR* (Fok1: rs2228570), *VDR* (Cdx-2: rs11568820), *VDR* (Taq1: rs731236), and *VDR* (BsmI: rs1544410) with lung cancer.

Comparison (model)	No.^a^	Sample size		Test of association				Test of heterogeneity			Publication bias
		Lung cancer	Ct^b^	OR	95% CI^d^	*p*-value	Model^e^ ^,^ ^f^	Q-test	*p*-value	I^b^ (%)	*p*-value (Egger’s)
*CYP24A1* (rs4809957)											
Allelic	2	1,612	2,112	1.048	0.909–1.208	0.521	F	0.094	0.759	0	NA
Homozygous		429	505	1.788	1.172–2.727	**0.007**	F	2.308	0.129	56.679	NA
Heterozygous		620	796	0.751	0.599–0.942	**0.013**	F	0.153	0.695	0	NA
Dominant		806	1,056	0.840	0.673–1.047	0.121	F	0.125	0.723	0	NA
Recessive		806	1,056	1.692	0.755–3.792	0.202	R	7.679	0.006	86.977	NA
*CYP3A4* (rs2740574)											
Allelic	4	3,504	4,182	1.269	1.053–1.530	**0.012**	F	1.898	0.594	0	0.57276
Homozygous	2	1,524	1859	1.465	0.903–2.377	0.122	F	0.007	0.933	0	NA
Heterozygous	4	1,705	2042	1.316	1.*043*–1.661	**0.021**	F	2.78	0.427	0	0.512
Dominant		1,752	2091	1.322	1.054–1.658	**0.016**	F	2.711	0.438	0	0.54792
Recessive	2	1,752	2091	1.149	0.741–1.782	0.535	F	0.049	0.825	0	NA
*VD*R (Fok1: rs2228570)											
Allelic	5	3,522	4,204	0.896	0.756–1.062	0.207	R	10.318	0.035	61.232	0.771
Homozygous		974	1,104	0.850	0.610–1.186	0.339	R	8.456	0.076	52.695	0.982
Heterozygous		1,465	1,746	0.858	0.744–0.988	**0.034**	F	5.237	0.264	23.627	0.522
Dominant		1,761	2,102	0.862	0.755–0.986	**0.030**	F	7.487	0.112	46.572	0.709
Recessive		1,761	2,102	0.961	0.808–1.42	0.649	F	6.893	0.142	41.967	0.948
*VDR* (Cdx-2: rs11568820)											
Allelic	3	2,904	3,424	0.907	0.8–1.027	0.125	F	3.369	0.185	40.643	0.725
Homozygous		885	987	0.828	0.535–1.281	0.397	F	3.363	0.186	40.535	0.094
Heterozygous		1,375	1,626	0.818	0.683–0.978	**0.028**	F	0.094	0.954	0	0.409
Dominant		1,452	1,712	0.807	0.676–0.962	**0.017**	F	0.042	0.979	0	0.835
Recessive		1,452	1,712	0.872	0.438–1.736	0.697	R	5.224	0.073	61.718	0.145
*VDR* (Taq1: rs731236)											
Allelic	5	3,662	4,404	0.89	0.804–0.986	**0.025**	F	6.022	0.198	33.576	0.103
Homozygous		1,140	1,335	0.776	0.618–0.976	**0.030**	F	4.924	0.295	18.763	0.767
Heterozygous		1,666	1,945	0.852	0.657–1.105	0.227	R	9.239	0.055	56.706	0.132
Dominant		1,831	2,202	0.891	0.774–1.026	0.11	F	7.713	0.103	48.138	0.103
Recessive		1,831	2,202	0.795	0.643–0.984	**0.035**	F	4.915	0.296	18.617	0.979
*VDR* (BsmI: rs1544410)											
Allelic	6	3,752	4,512	0.724	0.543–0.964	**0.027**	R	31.501	<0.001	84.128	0.135
Homozygous		1,158	1,357	0.701	0.488–1.007	0.055	R	25.95	<0.001	80.732	0.106
Heterozygous		1,690	1,940	0.753	0.529–1.07	0.114	R	21.379	0.001	76.613	0.069
Dominant		1,876	2,256	0.701	0.488–1.007	**0.055**	R	25.95	<0.001	80.732	0.106
Recessive		1,876	2,256	0.684	0.473–0.988	**0.043**	R	12.384	0.03	59.625	0.512

^a^ No.: no. of studies involved.

^b^ ^−ΔΔCT^.: control subjects.

^c^ OR: odds ratio.

^d^ 95% CI: 95% confidence interval.

^e^ R: random-effects model.

^f^ F: fixed-effects model; bold values indicate significance.

**FIGURE 3:**
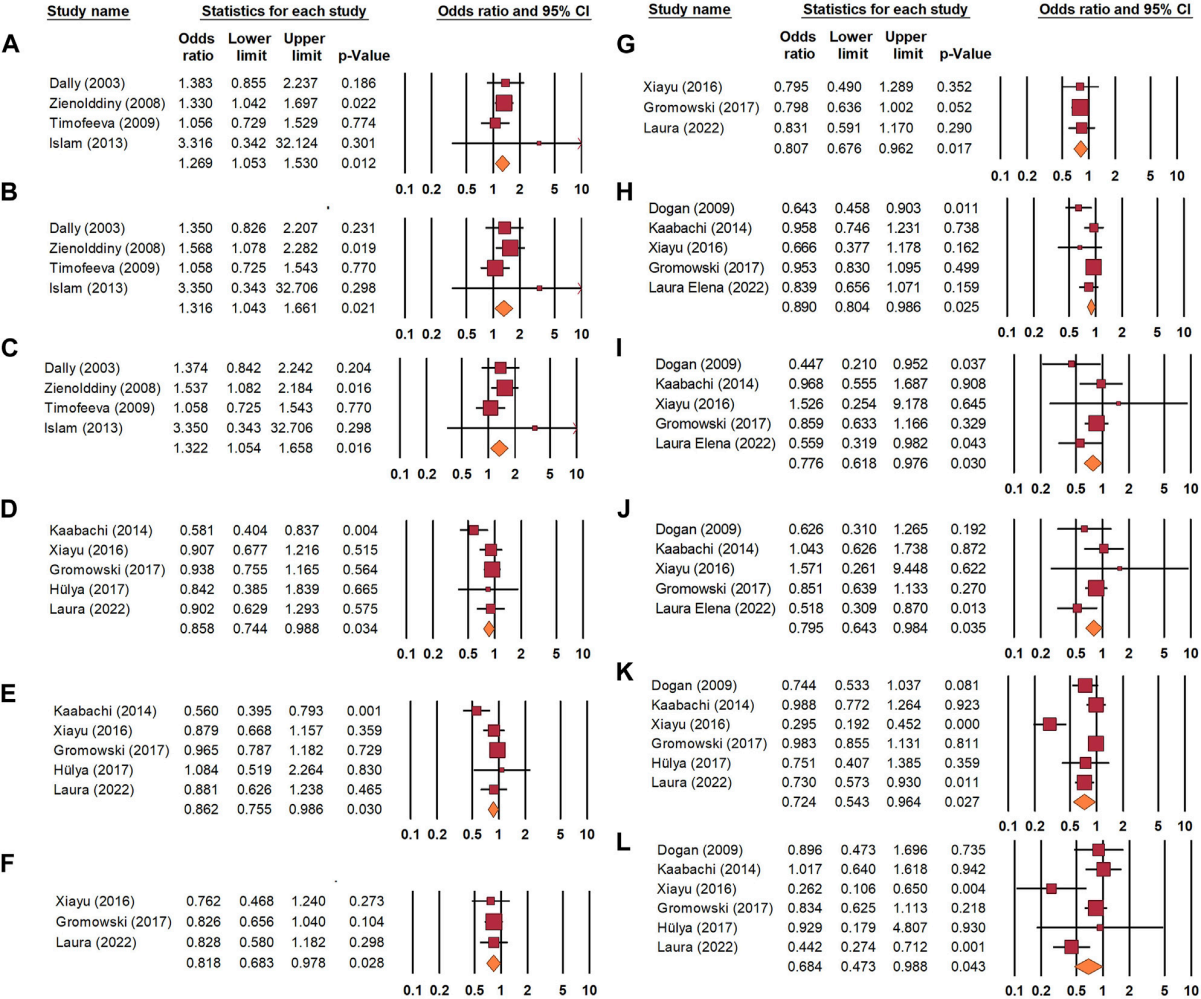
Association between *CYP3A4* (rs2740574) and lung cancer risk under **(A)** allelic, **(B)** heterozygous, and **(C)** dominant models. Association between *VDR* (Fok1: rs2228570) and decreased lung cancer risk under **(D)** heterozygous and **(E)** dominant models. Association between VDR (Cdx-2: rs11568820) and decreased lung cancer risk under **(F)** heterozygous and **(G)** dominant models. Association between *VDR* (Taq1: rs731236) and decreased lung cancer risk under **(H)** allelic, **(I)** homozygous, and **(J)** recessive models. Association between *VDR* (BsmI: rs1544410) and decreased lung cancer risk under **(K)** allelic and **(L)** recessive models.

Regarding *CYP3A4* (rs2740574), the meta-analysis addressed the risk association with lung cancer in the allelic [OR = 1.269, 95% CI 1.053–1.530, *p*-value = 0.012], heterozygous [OR = 1.316, 95% CI 1.043–1.661, *p*-value = 0.021], and dominant models [OR = 1.322, 95% CI 1.054–1.658, *p*-value = 0.016] (Figures 3A 3B,C).

*VDR* (Fok1: rs2228570) indicated a protective impact within the heterozygous model [OR = 0.858, 95% CI = 0.744–0.988, *p*-value = 0.034] (Figure 3D), and similar protective findings were found in the dominant model [OR = 0.862, 95% CI = 0.755–0.986, *p*-value = 0.030] (Figure 3E). *VDR* (Cdx-2: rs11568820) was found to be associated with protection from lung cancer under the heterozygous model [OR = 0.818, 95% CI = 0.683–978, *p*-value = 0.028] (Figure 3F), along with a positive impact of the variant under the dominant model [OR = 0.807, 95% CI = 0.676–0.962, *p*-value = 0.017] (Figure 3G).

*VDR* (Taq1: rs731236) exercised a protective role through our pooled analysis among allelic [OR = 0.89, 95% CI 0.804–0.986, *p*-value = 0.025], homozygous [OR = 0.776, 95% CI 0.618–0.976, *p*-value = 0.030], and recessive models [OR = 0.795, 95% CI 0.643–0.984, *p*-value = 0.035] (Figures 3H–J).

*VDR* (BsmI: rs1544410) reduced lung cancer risk in the allelic model [OR = 0.724, 95% CI, *p*-value], and a similar impact was noticed in the recessive model [OR = 0.684, 95% CI, *p*-value = 0.043] (Figure 3K and 3L) (Table 2).

Conversely, this meta-analysis revealed the lack of association of *CYP2R1* (rs10741657), *CYP27B1* (rs3782130), *CYP27B1* (rs10877012), *CYP24A1* (rs6068816), *CYP24A1* (rs4809960), *CYP3A5* (rs776746), *GC* (rs7041), *GC* (rs4588), and *VDR* (ApaI: rs7975232) with lung cancer [*p*-value >0.05] (Supplementary Table S3).

### 3.3 Heterogeneity analysis

The random-effects model was employed for the analysis to nullify the heterogeneity among studies under the recessive model for *CYP24A1* (rs4809957) and *CYP27B1* (rs10877012) (Table 2 and Supplementary Table S3. *CYP24A1* (rs6068816), *CYP24A1* (rs4809960), and *GC* (rs7041) were subjected to the random-effects model for all genetic models (Supplementary Table S3), as was *VDR* (BsmI: rs1544410). *CYP3A5* (rs776746) in both homozygous and recessive models was subjected to the random-effects model (Supplementary Table S3). *VDR* (ApaI: rs7975232) heterogeneity was observed in allelic, homozygous, and recessive models (Supplementary Table S3). Lastly, *VDR* (Cdx-2*:* rs11568820) and *VDR* (Taq1: rs731236) showed significant heterogeneity within the recessive and heterozygous models, respectively (Table 2).

### 3.4 Sensitivity analysis and publication bias

Sensitivity analysis was subsequently executed to evaluate whether our results were substantially affected by one individual study. Each time, we removed one included study to recalculate the significance of the results, and the obtained results showed that the ORs were not significantly changed. The graphs of Begg’s funnel were symmetrically executed, guaranteeing the absence of publication bias among the pooled studies in our meta-analysis (Supplementary Figure S2). Additionally, Egger’s regression test showed a *p*-value of >0.05 within included studies under all genetic models, addressing the lack of publication bias across our present study.

### 3.5 Genetic association models of the one-study variants with risk of lung cancer within included studies among *CYP27B1*, *CYP24A1*, *CYP3A4*, *GC*, and *VDR* with lung cancer polymorphisms in lung cancer risk

As depicted in Table 3, we subjected data of one-study variants to five heredity models to assess their association with lung cancer. Of the *CYP24A1*-included variants, *CYP24A1* (rs2585439) indicated significance with increased risk for lung cancer under allelic, homozygous, and recessive models [*p*-value <0.05]. Interestingly, three variants of *CYP24A1* showed an increased susceptibility to lung cancer within all genetic models, including rs2762937, rs2762940 and, rs2209314 [*p*-value <0.05]. Conversely, *CYP24A1* (rs6022993), *CYP24A1* (rs8120563), and *CYP24A1* (rs6068816) revealed a protective role for lung cancer in all genetic models [*p*-value <0.05]. *CYP24A1* (rs8120563) was correlated with increased lung cancer risk in allelic, heterozygous, and dominant models [*p*-value <0.05], while *CYP24A1* (rs2181874) was associated with lung cancer under the allelic model. Lastly, *CYP24A1* (rs6022999) indicated significance with lung cancer susceptibility under allelic and homozygous models (Table 3).

**TABLE 3:** Genetic association models of one-study variants with the risk of lung cancer within the included studies.

Study	Variant	Case (N)	Ct. (N)	Allelic model			Homozygous model			Heterozygous model			Dominant model			Recessive model		
				OR	95% CI	*p*-value	OR	95% CI	*p*-value	OR	95% CI	*p*-value	OR	95% CI	*p*-value	OR	95% CI	*p*-value
**Laura (2022)**	**rs4646536**	203	406	0.919	0.698–1.209	0.557	0.894	0.465–1.716	0.736	0.896	0.624–1.285	0.55	0.895	0.637–1.259	0.525	0.933	0.493–1.765	0.83
	**rs703842**	203	398	1.041	0.790–1.371	0.777	1.21	0.624–2.344	0.573	0.969	0.675–1.391	0.865	1.006	0.715–1.416	0.973	1.224	0.641–2.338	0.540
**Majda (2018)**	**rs2585439**	366	398	**1.277**	**1.027–1.587**	**0.028**	**2.122**	**1.292–3.484**	**0.003**	0.966	0.714–1.308	0.824	1.137	0.856–1.511	0.375	**2.155**	**1.337–3.472**	**0.002**
	**rs3787555**	364	391	0.919	0.720–1.172	0.495	0.669	0.355–1.260	0.213	1.037	0.761–1.412	0.819	0.971	0.724–1.303	0.846	0.660	0.354–1.233	0.193
	**rs3787557**	365	390	0.932	0.677–1.282	0.665	1.048	0.334–3.287	0.936	0.902	0.628–1.295	0.577	0.912	0.643–1.296	0.609	1.070	0.342–3.347	0.908
	**rs2762937**	364	382	**1.496**	**1.137–1.968**	**0.004**	**3.293**	**1.341–8.084**	**0.009**	**1.389**	**1.002–1.924**	**0.048**	**1.493**	**1.091–2.044**	**0.012**	**2.625**	**1.075–6.405**	**0.034**
	**rs6022993**	374	400	**0.383**	**0.260–0.566**	**< 0.001**	**0.168**	**0.037–0.766**	**0.021**	**0.414**	**0.269–0.639**	**<0.001**	**0.383**	**0.252–0.582**	**< 0.001**	**0.190**	**0.042–0.864**	**0.032**
	**rs8120563**	358	350	**0.628**	**0.399–0.988**	**0.044**	0.923	0.057–14.83	0.955	**0.596**	**0.370–0.962**	**0.034**	**0.603**	**0.376–0.967**	**0.036**	0.978	0.061–15.691	0.987
	**rs10623012**	381	401	1.190	0.975–1.451	0.086	1.378	0.942–2.016	0.099	0.868	0.618–1.219	0.414	1.032	0.754–1.413	0.843	**1.504**	**1.095–2.066**	**0.012**
	**rs2762940**	381	398	**1.687**	**1.318–2.160**	**< 0.001**	**3.113**	**1.596–6.076**	**0.001**	**1.522**	**1.116–2.077**	**0.008**	**1.693**	**1.261–2.274**	**< 0.001**	**2.715**	**1.402–5.258**	**0.003**
	**rs2762933**	377	399	**1.342**	**1.076–1.673**	**0.009**	**2.536**	**1.475–4.358**	**0.001**	1.033	0.767–1.392	0.831	1.213	0.915–1.608	0.18	**2.5**	**1.478–4.230**	**0.001**
	**rs2209314**	368	397	**1.584**	**1.229–2.042**	**< 0.001**	**2.933**	**1.367–6.292**	**0.006**	**1.486**	**1.088–2.029**	**0.013**	**1.607**	**1.192–2.167**	**0.002**	**2.58**	**1.211–5.497**	**0.014**
**Xiayu Wu (2016)**	**rs6068816**	426	445	**0.550**	**0.453–0.667**	**< 0.001**	**0.245**	**0.149–0.403**	**< 0.001**	**0.323**	**0.235–0.446**	**< 0.001**	**0.31**	**0.227–0.425**	**< 0.001**	**0.536**	**0.345–0.833**	**0.006**
	**rs2244719**	426	445	0.87	0.678–1.115	0.271	0.675	0.398–1.144	0.144	1.049	0.751–1.456	0.778	0.935	0.694–1.26	0.66	0.668	0.396–1.127	0.130
	**rs2762939**	426	445	0.987	0.815–1.195	0.892	1.046	0.704–1.553	0.825	0.851	0.635–1.141	0.281	0.897	0.681–1.183	0.443	1.146	0.8–1.64	0.458
	**rs2181874**	426	445	**1.325**	**1.031–1.703**	**0.028**	1.509	0.911–2.5	0.110	1.24	0.877–1.754	0.224	1.314	0.971–1.78	0.077	1.446	0.877–2.384	0.149
	**rs2296241**	426	445	0.858	0.711–1.036	0.112	0.709	0.48–1.049	0.085	0.971	0.708–1.331	0.853	0.89	0.658–1.2	0.44	0.723	0.52–1.007	0.055
**Qiantao (2020)**	**rs2585428**	550	800	1.073	0.920–1.251	0.370	1.145	0.846–1.551	0.379	1.155	0.886–1.505	0.288	1.151	0.898–1.477	0.267	1.043	0.814–1.337	0.739
	**rs6022999**	550	800	**1.296**	**1.093–1.537**	**0.003**	**2.014**	**1.354–2.997**	**0.001**	1.102	0.874–1.388	0.412	1.232	0.991–1.531	0.06	**1.934**	**1.316–2.843**	**0.001**
**Zhuoqi (2020)**	**rs3735451**	506	502	0.984	0.814–1.189	0.867	1.124	0.721–1.751	0.606	0.876	0.675–1.136	0.317	0.916	0.715–1.172	0.484	1.196	0.781–1.832	0.409
	**rs4646440**	506	503	**1.652**	**1.313–2.078**	**< 0.001**	**2.638**	**1.348–5.163**	**0.005**	**1.613**	**1.22–2.131**	**0.001**	**1.712**	**1.311–2.236**	**< 0.001**	**2.292**	**1.177–4.461**	**0.015**
	**rs35564277**	507	503	0.889	0.628–1.257	0.505	1.297	0.289–5.831	0.734	0.84	0.576–1.227	0.368	0.860	0.595–1.244	0.424	1.325	0.295–5.952	0.713
	**rs4646437**	507	505	**0.578**	**0.457–0.732**	**< 0.001**	0.661	0.321–1.363	0.262	**0.483**	**0.364–0.64**	**< 0.001**	**0.498**	**0.380–0.654**	**< 0.001**	0.815	0.397–1.672	0.577
**Majda (2018)**	**rs4237855**	368	398	1.229	0.996–1.518	0.055	**1.654**	**1.049–2.607**	**0.03**	1.074	0.792–1.456	0.645	1.184	0.889–1.576	0.247	**1.596**	**1.039–2.451**	**0.033**
	**rs2853559**	362	392	1.044	0.84–1.297	0.7	1.064	0.659–1.72	0.799	1.062	0.784–1.438	0.699	1.062	0.798–1.414	0.679	1.035	0.654–1.638	0.882
	**rs2239184**	363	387	1.213	0.988–1.489	0.065	1.324	0.88–1.99	0.178	**1.598**	**1.154–2.212**	**0.005**	**1.511**	**1.115–2.047**	**0.008**	1.012	0.705–1.452	0.951
	**rs2107301**	354	359	1.060	0.827–1.358	0.646	0.649	0.346–1.215	0.176	**1.424**	**1.033–1.963**	**0.031**	1.251	0.925–1.691	0.146	0.574	0.31–1.065	0.078
	**rs4760658**	365	359	0.956	0.762–1.199	0.695	0.882	0.52–1.497	0.643	0.985	0.728–1.334	0.924	0.966	0.726–1.285	0.810	0.888	0.531–1.484	0.65
	**rs6580642**	374	399	0.788	0.587–1.058	0.113	0.831	0.332–2.081	0.693	**0.690**	**0.489–0.976**	**0.036**	0.724	0.520–1.008	0.056	1.190	0.478–2.963	0.708
	**rs7967152**	375	399	1.182	0.966–1.446	0.104	1.270	0.851–1.895	0.242	**1.561**	**1.132–2.152**	**0.007**	**1.467**	**1.086–1.981**	**0.012**	0.98	0.689–1.396	0.912
	**rs7974353**	374	399	0.863	0.657–1.136	0.294	1.075	0.509–2.27	0.849	0.767	0.549–1.071	0.119	0.803	0.586–1.101	0.173	1.149	0.547–2.414	0.714
	**rs10875693**	366	395	**1.334**	**1.067–1.669**	**0.012**	1.245	0.772–2.007	0.368	**1.731**	**1.269–2.361**	**0.001**	**1.602**	**1.202–2.135**	**0.001**	1.002	0.632–1.59	0.992
	**rs7974708**	371	399	**1.273**	**1.021–1.588**	**0.032**	1.115	0.69–1.8	0.657	**1.714**	**1.261–2.328**	**0.001**	**1.556**	**1.170–2.069**	**0.002**	0.894	0.563–1.419	0.634
	**rs11574101**	370	395	1.074	0.742–1.554	0.707	0.726	0.225–2.063	0.548	1.241	0.801–1.921	0.333	1.152	0.765–1.733	0.498	0.707	0.249–2.006	0.515
	**rs2853563**	382	402	0.957	0.67–1.368	0.811	1.248	0.377–4.129	0.717	0.899	0.6–1.347	0.606	0.926	0.628–1.365	0.699	1.267	0.383–4.186	0.698
	**rs4760733**	362	377	1.176	0.958–1.444	0.12	1.324	0.879–1.994	0.18	**1.482**	**1.057–2.077**	**0.023**	**1.431**	**1.042–1.967**	**0.027**	1.036	0.730–1.472	0.842
	**rs10783218**	368	389	0.967	0.683–1.370	0.851	1.069	0.214–5.337	0.935	1.069	0.725–1.576	0.736	1.069	0.731–1.564	0.731	0.525	0.130–2.113	0.364
**Jinyu (2015)**	**rs11574129**	603	661	1.037	0.86–1.25	0.703	1.239	0.498–3.085	0.645	1.034	0.825–1.295	0.773	1.040	0.832–1.3	0.729	1.222	0.493–3.027	0.665

Bold values indicate significance.

Regarding the *CY3A4* gene, *CYP3A4* (rs4646440) indicated substantial significance with the risk of lung cancer within all genetic models [*p*-value <0.05]. *CYP3A4* (rs4646437) revealed a decreased risk under allelic, heterozygous, dominant, and recessive models [*p*-value <0.05] (Table 3).

*VDR* (rs4237855) and *VDR* (rs2107301) showed a significant risk for lung cancer in homozygous and heterozygous models, respectively. *VDR* (rs2239184), *VDR* (rs7967152), and *VDR* (rs4760733) were correlated with lung cancer susceptibility among heterozygous and dominant models [*p*-value <0.05] (Table 3). Both *VDR* (rs10875693) and *VDR* (rs7974708) showed a risk for lung cancer within allelic, heterozygous, and dominant genetic models [*p*-value <0.05]. *VDR* (rs6580642) had a protective impact within the heterozygous model (Table 3).

## 4 Discussion

Lung cancer is a critical problem for human health and is associated with a high rate of mortality. Therefore, its rapid and personalized management is an urgent priority. The well-established roles of vitamin D and genetic variants in the pathogenesis of several types of cancer, including lung cancer, are multifactorial and involve the alteration of transcription, translation, expression, and protein function (Shaw, 2013; Wang et al., 2017; Elsalahaty et al., 2023). Vitamin D has been attributed a favorable role in pulmonary inflammation, and its alteration was thought to be linked to lung cancer progression by modulating the tumor microenvironment and immune function (Prietl et al., 2013). Furthermore, vitamin D metabolites have been found to possess anticancer potency in preclinical models of lung cancer (Shaurova et al., 2018).

While numerous clinical trials have investigated the association of vitamin D gene variants with lung cancer, their findings were not conclusive and were not comprehensively analyzed through different genetic models. Additionally, the most recent studies have not been included. To the best of our knowledge, this is the first systematic review to extract all available data of vitamin D gene variants and subject them to genetic analyses. Our meta-analysis evidence is based on a sum of 6,206 lung cancer cases together with 7,272 healthy controls included in 16 case–control studies among seven genes of the vitamin D pathway (*CYP2R1*, *CYP27B1*, *CYP24A1*, *CYP3A4*, *CYP3A5*, *GC*, and *VDR*) to assess their association with lung cancer.

Our analysis showed a significant association of *CYP24A1* (rs4809957) with an increased risk of lung cancer in the homozygous model and a protective impact within the heterozygous comparison. However, this analysis is limited because of its dependence on data from two available studies. Altered levels of toxins manipulated through the *CYP450* machinery, including *CYP3A4* and *CYP3A5*, are thought to participate in the pathogenesis of lung cancer. Our analysis revealed that *CYP3A4* (rs2740574) has a significant association with increased lung cancer risk, while *CYP3A5* (rs776746) was not correlated with lung cancer risk.

Vitamin D-binding protein encoded by the *GC* gene is the main transport protein for 25(OH)D and vitamin D metabolites (Speeckaert et al., 2006; Anic et al., 2014). The potential implementation of *GC* in diseases is thought to be linked to the altered expression of 25(OH)D, actin scavenging, and immunomodulatory roles in macrophage activation and neutrophil chemotaxis (Chun, 2012). Our analysis could not reject the null hypothesis regarding *GC* (rs7041) and *GC* (rs4588), and no correlation was observed with the risk of lung cancer in all genetic models. These findings were consistent with those of a previous meta-analysis (Duan et al., 2020).

*VDR* is tightly linked to vitamin D levels attributed to the modulation of the risk of lung cancer due to the antiproliferative impact of vitamin D. *VDR* (Apa1: rs7975232) did not reveal a significant impact on lung cancer susceptibility. The same findings were confirmed in a recent meta-analysis (Li et al., 2019; Duan et al., 2020). However, *VDR* (Fok1: rs2228570) and *VDR* (Cdx-2: rs11568820) showed significant protection in heterozygous and dominant models. Similarly, Li et al. (2019) obtained a protective impact of Cdx-2: rs11568820 in heterozygous and dominant models relying on two reports. In addition, VDR (Taq1: rs731236) indicated a significant association with a decreased risk of lung cancer in allelic, homozygous, and recessive models. Lastly, *VDR* (BsmI: rs1544410) indicated protection against lung cancer in the allelic and recessive models.

In contrast, *CYP2R1* (rs10741657), *CYP27B* (rs3782130), *CYP27B1* (rs10877012), and *CYP24A1* (rs6068816) were not found to be associated with lung cancer. In agreement with Zhu et al. (2019), they did not show a clear association between *CYP27B1* (rs10877012) and cancer risk.

Our comprehensive systematic review and data extraction for all included variants, gathered data for one-study variants that were subjected to genetic models. As a result, *CYP24A1* (rs2762937), *CYP24A1* (rs2762940), and *CYP24A1* (rs2209314) showed a correlation with lung cancer susceptibility. In contrast, *CYP24A1* (rs6022993), *CYP24A1* (rs8120563), and *CYP24A1* (rs6068816) revealed a protective role for lung cancer in all genetic models. Our evidence-based study suggests that *CYP3A4* (rs2740574) contributes to the development of lung cancer by modulating vitamin D metabolism. It could therefore represent an independent risk factor for developing lung cancer. Genetic testing for this variant in lung cancer patients may be a useful tool for addressing which patients are characterized by higher aggressive characteristics and poorer outcomes, allowing clinicians to choose more personalized and robust treatments early in the course of the disease when treatment is most likely to be effective.

## 5 Conclusion

This systematic review and meta-analysis addressed the correlation of the *CYP3A4* (rs2740574) variant with lung cancer risk. Conversely, *VDR* (Fok1: rs2228570) and *VDR* (Cdx-2: rs11568820, Taq1: rs731236, and BsmI: rs1544410) showed protective effects. However, *CYP2R1* (rs10741657), *CYP27B1* (rs3782130), *CYP27B1* (rs10877012), *CYP24A1* (rs6068816), *CYP24A1* (rs4809960), *CYP3A5* (rs776746), *GC* (rs7041), *GC* (rs4588), and *VDR* (ApaI: rs7975232) did not show any significant association with lung cancer. Further assessment of vitamin D pathway genes with lung cancer is required, especially for *CYP3A4*, *CYP27B1*, *CYP24A1*, *GC*, and *VDR*, among different ethnicities, to generalize these associations.

## Data Availability

The original contributions presented in the study are included in the article/Supplementary Material; further inquiries can be directed to the corresponding author.
