# Supplementary material for: Revealing the association between vitamin D metabolic pathway gene variants and lung cancer risk: a systematic review and meta-analysis
Source: Front Genet. 2024 Feb 28;15:1302527. doi: 10.3389/fgene.2024.1302527 (PMC10933101; doi:10.3389/fgene.2024.1302527)
Supplement: Supplementary file 1 [file Table1.docx]

**Supplementary material**

**Revealing the association between vitamin D metabolic pathway gene variants and lung cancer risk: a systematic review and meta-analysis**

**Mohamed I. Elsalahaty, Samar Sami Alkafaas, Aya O. Bashir, Khaled A. El-Tarabily, Mohamed T. El-Saadony and Eman H. Yousef**

**Correspondence: Khaled A. El-Tarabily. (ktarabily@uaeu.ac.ae)**

| **Table S1.** Quality assessment criteria for included studies. | |  |
| --- | --- | --- |
| **Criteria** | **Score** |  |
| **1.Representativeness of cases** |  |  |
| Selected from cancer registry or multiple cancer center sites | 2 |  |
| Selected from oncology department or cancer institute | 1 | 1 |
| Not described | 0 |  |
| **2. Control source** |  |  |
| Population or community based | 2 |  |
| Hospital-based (cancer-free controls without diseases) | 1.5 | 1.5 |
| Healthy volunteers without total description | 1 |  |
| Cancer-free controls with other system diseases | 0.5 |  |
| Not described | 0 |  |
| **3.Ascertainment of cancer** |  |  |
| Histopathologic confirmation | 2 | 2 |
| Patient medical record | 1 |  |
| Not described | 0 |  |
| **4.Sample size** |  |  |
| >1000 | 2 |  |
| 200-1000 | 1 | 1 |
| <200 | 0 |  |
| **5.Quality control of genotyping methods** |  |  |
| Repetition of partial/total tested samples with a different method | 1 | 1 |
| Repetition of partial/total tested samples with the same method | 0.5 |  |
| Not described | 0 |  |
| **6.Hardy-Weinberg equilibrium (HWE)** |  |  |
| Hardy-Weinberg equilibrium in control subjects | 1 |  |
| Hardy-Weinberg disequilibrium in control subjects | 0 |  |

**Table S2.** Comprehensive data extraction for included studies.

| **Study ID** | **Variant** | **Cases** | | | | | **Control** | | | | | **M = /m=** | **Representativeness**  **of cases** | **Control**  **source** | **Ascertainment**  **of cancer** | **Sample size** | **Quality control of genotyping** | **P (HWE)**  **Control value/score** | **Score** |
| --- | --- | --- | --- | --- | --- | --- | --- | --- | --- | --- | --- | --- | --- | --- | --- | --- | --- | --- | --- |
|  |  | MM | Mm | Mm | M | m | MM | Mm | mm | M | m |  |  |  |  |  |  |  |  |
| Jinyu Kong (2015) | *CYP2R1* (rs10741657) | 252 | 270 | 80 | 774 | 430 | 246 | 326 | 89 | 818 | 504 | M=-G, m=A | 1 | 1.5 | 2 | 2 | 0.5 | 0.24/1 | 8 |
| Laura Elena (2022) | *CYP2R1* (rs10741657) | 78 | 97 | 26 | 253 | 149 | 156 | 172 | 70 | 484 | 312 | M=-G, m=A | 1 | 1.5 | 2 | 1 | 1 | 0.06/1 | 7.5 |
| Jinyu Kong (2015) | *CYP27B1*  (rs3782130) | 229 | 297 | 77 | 755 | 451 | 230 | 371 | 60 | 831 | 491 | M=C, m= G | 1 | 1.5 | 2 | 2 | 0.5 | 0/0 | 7 |
| Jinyu Kong (2015) | *CYP27B1*  (rs10877012) | 94 | 273 | 235 | 461 | 743 | 91 | 326 | 243 | 508 | 812 | M=G, m=T | 1 | 1.5 | 2 | 2 | 0.5 | 0.26/1 | 8 |
| Xiayu Wu (2016) | *CYP27B1*  (rs3782130) | 194 | 149 | 83 | 537 | 315 | 187 | 163 | 95 | 537 | 353 | M=C, m= G | 1 | 1.5 | 2 | 1 | 0.5 | <0.001/0 | 6 |
| Xiayu Wu (2016) | *CYP27B1*  (rs10877012) | 165 | 209 | 52 | 539 | 313 | 160 | 209 | 76 | 529 | 361 | M=G, m= T | 1 | 1.5 | 2 | 1 | 0.5 | 0.584/1 | 7 |
| Laura Elena (2022 | *CYP27B1*  (rs3782130) | 13 | 69 | 120 | 95 | 309 | 30 | 149 | 221 | 209 | 591 | M=C, m= G | 1 | 1.5 | 2 | 1 | 1 | 0.484/1 | 7.5 |
| Laura Elena (2022) | *CYP27B1*  (rs10877012) | 120 | 70 | 13 | 310 | 96 | 218 | 151 | 32 | 587 | 215 | M=G, m= T | 1 | 1.5 | 2 | 1 | 1 | 0.418/1 | 7.5 |
| Laura Elena (2022) | *CYP27B1* (rs4646536) | 118 | 70 | 15 | 306 | 100 | 225 | 149 | 32 | 599 | 213 | M=A, m= G | 1 | 1.5 | 2 | 1 | 1 | 0.297/1 | 7.5 |
| Laura Elena (2022) | *CYP27B1*  (rs703842) | 116 | 71 | 16 | 303 | 103 | 228 | 144 | 26 | 600 | 196 | M=T, m=C | 1 | 1.5 | 2 | 1 | 1 | 0.614/1 | 7.5 |
| Jinyu Kong (2015) | *CYP24A1*  (rs6068816) | 217 | 314 | 72 | 748 | 458 | 110 | 465 | 86 | 685 | 637 | M=C, m=T | 1 | 1.5 | 2 | 2 | 0.5 | <0.001/0 | 7 |
| Jinyu Kong (2015) | *CYP24A1*  (rs4809957) | 234 | 309 | 60 | 777 | 429 | 228 | 406 | 27 | 862 | 460 | M=G, m=A | 1 | 1.5 | 2 | 2 | 0.5 | <0.001/0 | 7 |
| Ruoyi Qu (2018) | *CYP24A1*  (rs6068816) | 160 | 155 | 30 | 475 | 215 | 131 | 178 | 42 | 440 | 262 | M=C, m=T | 2 | 1.5 | 2 | 1 | 0.5 | 0.12/1 | 8 |
| Qiantao Xiong (2020) | *CYP24A1*  (rs6068816) | 226 | 246 | 78 | 698 | 402 | 362 | 348 | 90 | 1072 | 528 | M=C, m=T | 2 | 1.5 | 2 | 2 | 0.5 | 0.65/1 | 9 |
| Qiantao Xiong (2020) | *CYP24A1*  (rs4809960) | 329 | 194 | 468 | 852 | 1130 | 468 | 290 | 42 | 1226 | 374 | M=T, m=C | 2 | 1.5 | 2 | 2 | 0.5 | 0.74/1 | 9 |
| Qiantao Xiong (2020) | *CYP24A1*  (rs2585428) | 135 | 271 | 144 | 541 | 559 | 218 | 379 | 203 | 815 | 785 | M=G, m=A | 2 | 1.5 | 2 | 2 | 0.5 | 0.14/1 | 9 |
| Qiantao Xiong (2020) | *CYP24A1*  (rs6022999) | 276 | 210 | 64 | 762 | 338 | 443 | 306 | 51 | 1192 | 408 | M=A, m=G | 2 | 1.5 | 2 | 2 | 0.5 | 0.85/1 | 9 |
| Laura Elena (2022) | *CYP24A1*  (rs6068816) | 152 | 44 | 7 | 348 | 58 | 325 | 75 | 3 | 725 | 81 | M=C, m=T | 1 | 1.5 | 2 | 1 | 1 | 0.5/1 | 7.5 |
| Laura Elena (2022) | *CYP24A1*  (rs4809957) | 9 | 68 | 126 | 86 | 320 | 17 | 145 | 233 | 179 | 611 | M=G, m=A | 1 | 1.5 | 2 | 1 | 1 | 0.35/1 | 7.5 |
| Xiayu Wu (2016) | *CYP24A1*  (rs6068816) | 349 | 62 | 15 | 760 | 92 | 360 | 75 | 10 | 795 | 95 | M=T, m=C | 1 | 1.5 | 2 | 1 | 0.5 | 0.014/0 | 6 |
| Xiayu Wu (2016) | *CYP24A1*  (rs2244719) | 170 | 222 | 34 | 562 | 290 | 76 | 307 | 62 | 459 | 431 | M=C, m=T | 1 | 1.5 | 2 | 1 | 0.5 | 0/0 | 6 |
| Xiayu Wu (2016) | *CYP24A1*  (rs4809960) | 312 | 89 | 25 | 713 | 139 | 320 | 87 | 38 | 727 | 163 | M=T, m=C | 1 | 1.5 | 2 | 1 | 0.5 | 0/0 | 6 |
| Xiayu Wu (2016) | *CYP24A1*  (rs2762939) | 160 | 192 | 74 | 512 | 340 | 156 | 220 | 69 | 532 | 358 | M=G, m=C | 1 | 1.5 | 2 | 1 | 0.5 | 0.55/1 | 7 |
| Xiayu Wu (2016) | *CYP24A1*  (rs2762939) | 303 | 84 | 39 | 690 | 162 | 340 | 76 | 29 | 756 | 134 | M=G, m=A | 1 | 1.5 | 2 | 1 | 0.5 | 0/0 | 6 |
| Xiayu Wu (2016) | *CYP24A1*  (rs2296241) | 119 | 230 | 77 | 468 | 384 | 114 | 227 | 104 | 455 | 435 | M=G, m=A | 1 | 1.5 | 2 | 1 | 0.5 | 0.66/1 | 7 |
| Majda Haznadar (2018) | *CYP24A1*  (rs2585439) | 174 | 139 | 53 | 487 | 245 | 202 | 167 | 29 | 571 | 225 | M=A, m=G | 2 | 2 | 2 | 1 | 0.5 | 0.49/1 | 7.5 |
| Majda Haznadar (2018) | *CYP24A1*  (rs3787555) | 225 | 122 | 17 | 572 | 156 | 239 | 125 | 27 | 603 | 179 | M=C, m=A | 2 | 2 | 2 | 1 | 0.5 | 0.06/1 | 7.5 |
| Majda Haznadar (2018) | *CYP24A1*  (rs3787557) | 291 | 68 | 6 | 650 | 80 | 305 | 79 | 6 | 689 | 91 | M=T, m=C | 2 | 2 | 2 | 1 | 0.5 | 0.73/1 | 7.5 |
| Majda Haznadar (2018) | *CYP24A1*  (rs2762937) | 238 | 109 | 17 | 585 | 143 | 282 | 93 | 7 | 657 | 107 | M=A, m=G | 2 | 2 | 2 | 1 | 0.5 | 0.83/1 | 7.5 |
| Majda Haznadar (2018) | *CYP24A1*  (rs6022993) | 338 | 34 | 2 | 710 | 38 | 313 | 76 | 11 | 702 | 98 | M=C, m=T | 2 | 2 | 2 | 1 | 0.5 | 0.02/0 | 6.5 |
| Majda Haznadar (2018) | *CYP24A1*  (rs8120563) | 326 | 31 | 1 | 683 | 33 | 301 | 48 | 1 | 650 | 50 | M=A, m=T | 2 | 2 | 2 | 1 | 0.5 | 0.53/1 | 7.5 |
| Majda Haznadar (2018) | *CYP24A1*  (rs10623012) | 104 | 158 | 119 | 366 | 396 | 112 | 196 | 93 | 420 | 382 | Deletion | 2 | 2 | 2 | 1 | 0.5 | 0.685/1 | 7.5 |
| Majda Haznadar (2018) | *CYP24A1*  (rs2762940) | 219 | 130 | 32 | 568 | 194 | 277 | 108 | 13 | 662 | 134 | M=A, m=C | 2 | 2 | 2 | 1 | 0.5 | 0.54/1 | 7.5 |
| Majda Haznadar (2018) | *CYP24A1*  (rs2762933) | 185 | 144 | 48 | 514 | 240 | 215 | 162 | 22 | 592 | 206 | M=T, m=A | 2 | 2 | 2 | 1 | 0.5 | 0.23/1 | 7.5 |
| Majda Haznadar (2018) | *CYP24A1*  (rs2209314) | 218 | 127 | 23 | 563 | 173 | 278 | 109 | 10 | 665 | 129 | M=T, m=C | 2 | 2 | 2 | 1 | 0.5 | 0.86/1 | 7.5 |
| Heike Dally (2003) | *CYP3A4* (rs2740574) | 723 | 58 | 1 | 1504 | 60 | 404 | 24 | 0 | 832 | 24 | M=A, m=G | 1 | 1.5 | 0 | 2 | 0 | 0.551/1 | 5.5 |
| Shanbeh Zienolddiny (2008) | *CYP3A4* (rs2740574) | 81 | 123 | 46 | 285 | 215 | 126 | 122 | 49 | 374 | 220 | M=A, m=G | 2 | 2 | 2 | 1 | 0.5 | 0.039/0 | 7.5 |
| Maria N.Timofeeva (2009) | *CYP3A4* (rs2740574) | 570 | 44 | 0 | 1184 | 44 | 1165 | 85 | 0 | 2415 | 85 | M=A, m=G | 2 | 2 | 2 | 2 | 0 | 0.213/1 | 9 |
| Mohammad Safiqul Islam (2013) | *CYP3A4* (rs2740574) | 103 | 3 | 0 | 209 | 3 | 115 | 1 | 0 | 231 | 1 | M=A, m=G | 2 | 1 | 2 | 1 | 0 | 0.963/1 | 7 |
| Zhuoqi Jia (2020) | *CYP3A4* (rs3735451) | 247 | 208 | 51 | 702 | 310 | 234 | 225 | 43 | 693 | 31 | M=T, m=C | 1 | 1 | 2 | 0 | 1 | 0.281/1 | 7 |
| Zhuoqi Jia (2020) | *CYP3A4* (rs4646440) | 312 | 165 | 29 | 789 | 223 | 369 | 121 | 13 | 859 | 147 | M=G, m=A | 1 | 1 | 2 | 0 | 1 | 0.419/1 | 7 |
| Zhuoqi Jia (2020) | *CYP3A4* (rs35564277) | 446 | 57 | 4 | 949 | 65 | 434 | 66 | 3 | 934 | 72 | M=T, m=C | 1 | 1 | 2 | 0 | 1 | 0.776/1 | 7 |
| Zhuoqi Jia (2020) | *CYP3A4* (rs4646437) | 386 | 107 | 14 | 879 | 135 | 310 | 178 | 17 | 798 | 212 | M=G, m=A | 1 | 1 | 2 | 0 | 1 | 0.159/1 | 7 |
| Heike Dally (2003) | *CYP3A5* (rs776746) | 680 | 92 | 10 | 1452 | 112 | 376 | 51 | 1 | 803 | 53 | M=G, m=A | 1 | 1.5 | 0 | 2 | 1 | 0.59/1 | 5.5 |
| Maria N.Timofeeva (2009) | *CYP3A5 (rs776746)* | 542 | 71 | 0 | 1155 | 71 | 1100 | 152 | 0 | 2352 | 152 | M=G, m=A | 2 | 2 | 2 | 2 | 0 | 0.02/0 | 8 |
| Mohammad Safiqul Islam (2013) | *CYP3A5* (rs776746) | 58 | 36 | 12 | 152 | 60 | 62 | 37 | 17 | 161 | 71 | M=G, m=A | 2 | 1 | 2 | 1 | 0 | 0.007/0 | 6 |
| Jinyu Kong (2015) | *GC* (rs7041) | 329 | 240 | 34 | 898 | 308 | 272 | 339 | 50 | 883 | 439 | M=T, m=G | 1 | 1.5 | 2 | 2 | 0.5 | <0.001/0 | 7 |
| Wanwisa Maneechay (2015) | *GC* (rs7041) | 42 | 61 | 10 | 145 | 81 | 58 | 47 | 8 | 163 | 63 | M=T, m=G | 1 | 1 | 0 | 1 | 1 | 0.715/1 | 5 |
| Wanwisa Maneechay (2015) | *GC* (rs4588) | 69 | 40 | 4 | 178 | 48 | 60 | 47 | 6 | 167 | 59 | M=C, m= A | 1 | 1 | 0 | 1 | 1 | 0.407/1 | 5 |
| Xiayu Wu (2016) | *GC* (rs7041) | 175 | 230 | 21 | 580 | 272 | 173 | 225 | 47 | 571 | 319 | M=T, m=G | 1 | 1.5 | 2 | 1 | 0.5 | 0.036/0 | 7 |
| Xiayu Wu (2016) | *GC* (rs4588) | 230 | 170 | 26 | 630 | 222 | 235 | 173 | 37 | 643 | 247 | M=C, m= A | 1 | 1.5 | 2 | 1 | 0.5 | 0.519/1 | 7 |
| Laura Elena (2022) | *GC* (rs7041) | 43 | 92 | 68 | 178 | 228 | 77 | 212 | 115 | 366 | 442 | M=T, m=G | 1 | 1.5 | 2 | 1 | 1 | 0.237/1 | 7.5 |
| Dogan (2009) | *VDR*  (ApaI: rs7975232) | 44 | 64 | 29 | 152 | 122 | 58 | 76 | 22 | 192 | 120 | M=A, m=C | 1 | 1.5 | 1 | 1 | 0 | 0.71/1 | 5.5 |
| Dogan (2009) | *VDR*  (Taq1: rs731236) | 64 | 59 | 14 | 187 | 87 | 49 | 83 | 24 | 181 | 131 | M=T, m=C | 1 | 1.5 | 1 | 1 | 0 | 0.25/1 | 5.5 |
| Dogan (2009) | *VDR*  (BsmI: rs1544410) | 57 | 60 | 20 | 174 | 100 | 45 | 86 | 25 | 176 | 136 | M=C, m=T | 1 | 1.5 | 1 | 1 | 0 | 0.13/1 | 5.5 |
| Kaabachi (2014) | *VDR*  (ApaI: rs7975232) | 101 | 118 | 21 | 320 | 160 | 100 | 134 | 46 | 334 | 226 | M=A, m=C | 1 | 1 | 2 | 1 | 0.5 | 0.92/1 | 6.5 |
| Kaabachi (2014) | *VDR*  (Fok1: rs2228570) | 134 | 90 | 16 | 358 | 122 | 116 | 134 | 30 | 366 | 194 | M=C, m=T | 1 | 1 | 2 | 1 | 0.5 | 0.34/1 | 6.5 |
| Kaabachi (2014) | *VDR*  (Taq1: rs731236) | 90 | 118 | 32 | 298 | 182 | 98 | 146 | 36 | 342 | 218 | M=T, m=C | 1 | 1 | 2 | 1 | 0.5 | 0.11/1 | 6.5 |
| Kaabachi (2014) | *VDR*  (BsmI: rs1544410) | 74 | 126 | 40 | 274 | 206 | 84 | 150 | 46 | 318 | 242 | M=C, m=T | 1 | 1 | 2 | 1 | 0.5 | 0.13/1 | 6.5 |
| Xiayu Wu (2016) | *VDR*  (ApaI: rs7975232) | 95 | 191 | 140 | 381 | 471 | 89 | 214 | 142 | 392 | 498 | M=A, m=C | 1 | 1.5 | 2 | 1 | 0.5 | 0.61/1 | 7 |
| Xiayu Wu (2016) | *VDR*  (Fok1: rs2228570) | 166 | 192 | 68 | 524 | 328 | 160 | 204 | 81 | 524 | 366 | M=C, m=T | 1 | 1.5 | 2 | 1 | 0.5 | 0.26/1 | 7 |
| Xiayu Wu (2016) | *VDR*  (Cdx-2: rs11568820) | 39 | 324 | 63 | 402 | 450 | 33 | 360 | 52 | 426 | 464 | M=C, m=T | 1 | 1.5 | 2 | 1 | 0.5 | 0/0 | 6 |
| Xiayu Wu (2016) | *VDR*  (Taq1: rs731236) | 409 | 14 | 3 | 832 | 20 | 416 | 27 | 2 | 859 | 31 | M=T, m=C | 1 | 1.5 | 2 | 1 | 0.5 | 0.04/0 | 6 |
| Xiayu Wu (2016) | *VDR*  (BsmI: rs1544410) | 403 | 17 | 6 | 823 | 29 | 373 | 49 | 23 | 795 | 95 | M=C, m=T | 1 | 1.5 | 2 | 1 | 0.5 | 0/0 | 6 |
| Gromowski (2017) | *VDR*  (ApaI: rs7975232) | 175 | 412 | 236 | 732 | 884 | 184 | 500 | 235 | 868 | 970 | M=A, m=C | 1 | 2 | 1 | 2 | 1 | 0.006/0 | 7 |
| Gromowski (2017) | *VDR*  (Fok1: rs2228570) | 258 | 395 | 180 | 911 | 755 | 277 | 452 | 188 | 1006 | 828 | M=C, m=T | 1 | 2 | 1 | 2 | 1 | 0.88/1 | 8 |
| Gromowski (2017) | *VDR*  (Cdx-2: rs11568820) | 649 | 170 | 3 | 1468 | 176 | 653 | 207 | 11 | 1513 | 229 | M=C, m=T | 1 | 2 | 1 | 2 | 1 | 0.23/1 | 8 |
| Gromowski (2017) | *VDR*  (Taq1: rs731236*)* | 340 | 390 | 95 | 1070 | 580 | 375 | 423 | 122 | 1173 | 667 | M=T, m=C | 1 | 2 | 1 | 2 | 1 | 0.87/1 | 8 |
| Gromowski (2017) | *VDR*  (BsmI: rs1544410) | 330 | 388 | 92 | 1048 | 572 | 384 | 410 | 122 | 1178 | 654 | M=C, m=T | 1 | 2 | 1 | 2 | 1 | 0.45/1 | 8 |
| Laura Elena (2022) | *VDR*  (ApaI: rs7975232) | 52 | 98 | 54 | 202 | 206 | 121 | 203 | 79 | 445 | 361 | M=A, m=C | 1 | 1.5 | 2 | 1 | 1 | 0.71/1 | 7.5 |
| Laura Elena (2022) | *VDR*  (Fok1: rs2228570) | 89 | 90 | 24 | 268 | 138 | 165 | 185 | 55 | 515 | 295 | M=C, m=T | 1 | 1.5 | 2 | 1 | 1 | 0.78/1 | 7.5 |
| Laura Elena (2022) | *VDR*  (Cdx-2: rs11568820) | 120 | 73 | 11 | 313 | 95 | 215 | 158 | 23 | 588 | 204 | M=C, m=T | 1 | 1.5 | 2 | 1 | 1 | 0.39/1 | 7.5 |
| Laura Elena (2022) | *VDR*  (Taq1: rs731236) | 72 | 110 | 21 | 254 | 152 | 140 | 188 | 73 | 72 | 110 | M=T, m=C | 1 | 1.5 | 2 | 1 | 1 | 0.48/1 | 7.5 |
| Laura Elena (2022) | *VDR*  (BsmI: rs1544410) | 71 | 108 | 25 | 250 | 158 | 126 | 181 | 97 | 433 | 375 | M=C, m=T | 1 | 1.5 | 2 | 1 | 1 | 0.045/0 | 6.5 |
| Hülya Kanbur (2017) | *VDR*  (Fok1: rs2228570) | 31 | 20 | 8 | 64 | 36 | 30 | 23 | 2 | 83 | 27 | M=C, m=T | 1 | 1 | 1 | 0 | 0 | 0.34/1 | 4 |
| Hülya Kanbur (2017) | *VDR*  (BsmI: rs1544410) | 37 | 19 | 3 | 93 | 25 | 29 | 23 | 3 | 81 | 29 | M=C, m=T | 1 | 1 | 1 | 0 | 0 | 0.57/1 | 4 |
| Jinyu Kong (2015) | *VDR* (rs11574129) | 339 | 254 | 10 | 932 | 274 | 378 | 274 | 9 | 1030 | 292 | M=T, m=C | 1 | 1.5 | 2 | 2 | 0.5 | 0/0 | 7 |
| Majda Haznadar (2018) | *VDR* (rs4237855) | 153 | 158 | 57 | 428 | 272 | 182 | 175 | 41 | 539 | 257 | M=A , r = G | 2 | 2 | 2 | 1 | 0.5 | 0.91/1 | 8.5 |
| Majda Haznadar (2018) | *VDR* (rs2853559) | 170 | 152 | 40 | 392 | 232 | 190 | 160 | 42 | 540 | 244 | M=C, m =T | 2 | 2 | 2 | 1 | 0.5 | 0.34/1 | 8.5 |
| Majda Haznadar (2018) | *VDR* (rs2239184) | 108 | 184 | 71 | 400 | 326 | 151 | 161 | 75 | 463 | 311 | M=T, m= C | 2 | 2 | 2 | 1 | 0.5 | 0.008/0 | 7.5 |
| Majda Haznadar (2018) | *VDR* rs2107301* | 207 | 130 | 17 | 544 | 161 | 229 | 101 | 29 | 559 | 231 | M=C, m =T | 2 | 2 | 2 | 1 | 0.5 | <0.001/0 | 7.5 |
| Majda Haznadar (2018) | *VDR* (rs4760658) | 200 | 136 | 29 | 536 | 194 | 213 | 147 | 35 | 573 | 217 | M=A , m = G | 2 | 2 | 2 | 1 | 0.5 | 0.189/1 | 8.5 |
| Majda Haznadar (2018) | *VDR* (rs6580642) | 294 | 70 | 10 | 658 | 90 | 290 | 100 | 9 | 680 | 118 | M=C, m =T | 2 | 2 | 2 | 1 | 0.5 | 0.912/1 | 8.5 |
| Majda Haznadar (2018) | *VDR* (rs7967152) | 110 | 191 | 74 | 411 | 339 | 151 | 168 | 80 | 470 | 328 | M=C, m =A | 2 | 2 | 2 | 1 | 0.5 | 0.009/0 | 7.5 |
| Majda Haznadar (2018) | *VDR* (rs7974353) | 278 | 81 | 15 | 637 | 111 | 279 | 106 | 14 | 664 | 134 | M=C, m =T | 2 | 2 | 2 | 1 | 0.5 | 0.325/1 | 8.5 |
| Majda Haznadar (2018) | *VDR* (rs10875693) | 176 | 151 | 39 | 503 | 229 | 236 | 117 | 42 | 589 | 201 | M=T, m= A | 2 | 2 | 2 | 1 | 0.5 | <0.001/0 | 7.5 |
| Majda Haznadar (2018) | *VDR* (rs7974708) | 175 | 159 | 37 | 509 | 233 | 232 | 123 | 44 | 587 | 211 | M=T, m= C | 2 | 2 | 2 | 1 | 0.5 | <0.001/0 | 7.5 |
| Majda Haznadar (2018) | *VDR* (rs11574101) | 315 | 49 | 6 | 679 | 61 | 343 | 43 | 9 | 729 | 61 | T, deletion | 2 | 2 | 2 | 1 | 0.5 | <0.001/0 | 7.5 |
| Majda Haznadar (2018) | *VDR* (rs2853563) | 325 | 51 | 6 | 701 | 63 | 338 | 59 | 5 | 735 | 69 | M=G, m=A | 2 | 2 | 2 | 1 | 0.5 | 0.195/1 | 8.5 |
| Majda Haznadar (2018) | *VDR* (rs4760733) | 94 | 189 | 79 | 377 | 347 | 126 | 171 | 80 | 423 | 331 | M=G, m=A | 2 | 2 | 2 | 1 | 0.5 | 0.124/1 | 8.5 |
| Majda Haznadar (2018) | *VDR* (rs10783218) | 304 | 61 | 3 | 669 | 67 | 325 | 61 | 3 | 711 | 73 | M=C, m =T | 2 | 2 | 2 | 1 | 0.5 | 0.119/1 | 8.5 |

**Table S3**. Meta-analysis of the association between *CYP2R1* (rs10741657), *CYP27B1* (rs3782130), *CYP27B1* (rs10877012), *CYP24A1* (rs6068816), *CYP24A1* (rs4809960), *CYP3A5* (rs776746), *GC* (rs7041), *GC* (rs4588), *VDR* (ApaI: rs7975232) with lung cancer*.*

| **Comparison (Model)** | **Number of studies** | **Sample size** | | **Test of association** | | | | | **Test of heterogeneity** | | | **Publication bias** |
| --- | --- | --- | --- | --- | --- | --- | --- | --- | --- | --- | --- | --- |
|  |  | **Lung cancer** | **Ct.** | **OR^2^** | **95% CI^1^** | **P-value** | **Model^3,4^** | | **Q test** | **P-value** | **I^2^ (%)** | **P-value**  **(Eggers)** |
| ***CYP2R1* (rs10741657)** | | | | | | | | | | | | |
| Allelic | **2** | 1606 | 2118 | 0.905 | 0.791- 1.037 | 0.15 | F | | 0.008 | 0.931 | 0 | NA |
| Homozygous |  | 436 | 561 | 0.834 | 0.623-1.115 | 0.221 | F | | 0.267 | 0.605 | 0 | NA |
| Heterozygous |  | 697 | 900 | 0.892 | 0.73-1.09 | 0.263 | F | | 2.210 | 0.137 | 54.750 | NA |
| Dominant |  | 803 | 1059 | 0.876 | 0.725-1.059 | 0.173 | F | | 0.992 | 0.319 | 0 | NA |
| Recessive |  | 803 | 1059 | 0.885 | 0.676-1.159 | 0.375 | F | | 1.355 | 0.244 | 26.17 | NA |
| ***CYP27B1* (rs3782130)** | | | | | | | | | | | | |
| Allelic | **3** | 2462 | 3012 | 0.989 | 0.884-1.108 | 0.854 | | F | 2.285 | 0.319 | 12.478 | 0.67 |
| Homozygous |  | 716 | 823 | 1.052 | 0.824-1.343 | 0.685 | | F | 2.82 | 0.244 | 29.083 | 0.717 |
| Heterozygous |  | 951 | 1130 | 0.847 | 0.707-1.014 | 0.070 | | F | 0.661 | 0.718 | 0 | 0.228 |
| Dominant |  | 1231 | 1506 | 0.886 | 0.749-1.049 | 0.161 | | F | 0.735 | 0.692 | 0 | 0.134 |
| Recessive |  | 1231 | 1506 | 1.142 | 0.937-1.392 | 0.188 | | F | 4.089 | 0.129 | 51.091 | 0.0672 |
| ***CYP27B1* (rs10877012)** | | | | | | | | | | | | |
| Allelic | ***3*** | 2462 | 3012 | 0.924 | 0.826-1.035 | 0.171 | | F | 2.228 | 0.328 | 10.245 | 0.463 |
| Homozygous |  | 679 | 820 | 0.805 | 0.63-1.028 | 0.083 | | F | 1.659 | 0.436 | 0 | 0.665 |
| Heterozygous |  | 931 | 1155 | 0.882 | 0.732-1.063 | 0.187 | | F | 0.724 | 0.696 | 0 | 0.377 |
| Dominant |  | 1231 | 1506 | 0.863 | 0.723-1.03 | 0.102 | | F | 0.112 | 0.945 | 0 | 0.142 |
| Recessive |  | 1231 | 1506 | 0.872 | 0.611-1.244 | 0.450 | | R | 4.957 | 0.084 | 59.652 | 0.49 |
| ***CYP24A1* (rs6068816)** | | | | | | | | | | | | |
| Allelic | 4 | 3402 | 4430 | 0.949 | 0.669-1.346 | 0.770 | R | | 34.71 | **< 0.001** | 91.357 | 0.618 |
| Homozygous |  | 942 | 1149 | 0.962 | 0.439-2.108 | 0.923 | R | | 28.277 | **< 0.001** | 89.391 | 0.706 |
| Heterozygous |  | 1514 | 1994 | 0.762 | 0.414-1.4.02 | 0.383 | R | | 50.179 | **< 0.001** | 94.021 | 0.906 |
| Dominant |  | 1701 | 2215 | 0.793 | 0.426-1.477 | 0.464 | R | | 56.851 | **< 0.001** | 94.723 | 0.958 |
| Recessive |  | 1701 | 2215 | 1.089 | 0.710-1.672 | 0.695 | R | | 9.743 | **0.021** | 69.208 | 0.555 |
| ***CYP24A1* (rs4809960)** | | | | | | | | | | | | |
| Allelic | 2 | 2834 | 2550 | 2.191 | 0.564-8.503 | 0.257 | R | | 65.082 | **< 0.001** | 98.463 | NA |
| Homozygous |  | 1161 | 880 | 5.105 | 0.522-49.877 | 0.161 | R | | 26.611 | **< 0.001** | 96.242 | NA |
| Heterozygous |  | 934 | 1193 | 0.923 | 0.759-1.121 | 0.418 | F | | 0.246 | 0.620 | 0 | NA |
| Dominant |  | 1417 | 1245 | 1.643 | 0.554-4.878 | 0.371 | R | | 30.75 | **< 0.001** | 96.748 | NA |
| Recessive |  | 1417 | 1245 | 5.221 | 0.538-50.662 | 0.154 | R | | 26.827 | **< 0.001** | 96.272 | NA |
| ***CYP3A5* (rs776746)** | | | | | | | | | | | | |
| Allelic | 3 | 3003 | 3592 | 1.004 | 0.827-1.219 | 0.969 | F | | 1.204 | 0.548 | 0 | 0.912 |
| Homozygous | 2 | 1302 | 1556 | 1.618 | 0.243-10.791 | 0.619 | R | | 3.1 | **0.078** | 67.741 | NA |
| Heterozygous | 3 | 1479 | 1778 | 0.977 | 0.788-1.212 | 0.834 | F | | 0.096 | 0.953 | 0 | 0.273 |
| Dominant |  | 1501 | 1796 | 0.994 | 0.805-1.227 | 0.954 | F | | 0.353 | 0.838 | 0 | 0.987 |
| Recessive | 2 | 1501 | 1796 | 1.604 | 0.237-10.858 | 0.628 | R | | 3.18 | **0.075** | 68.55 | NA |
| ***GC* (rs7041)** | | | | | | | | | | | | |
| Allelic | 4 | 2690 | 3246 | 0.934 | 0.714-1.220 | 0.615 | R | | 15.721 | **0.001** | 80.917 | 0.07024 |
| Homozygous |  | 722 | 800 | 0.753 | 0.450-1.260 | 0.280 | R | | 9.418 | **0.024** | 68.145 | 0.54889 |
| Heterozygous |  | 1212 | 1403 | 0.914 | 0.598-1.398 | 0.679 | R | | 18.019 | **<0.001** | 83.350 | 0.28545 |
| Dominant |  | 1345 | 1623 | 0.910 | 0.609-1.361 | 0.647 | R | | 17.496 | **0.001** | 82.584 | 1.4555 |
| Recessive |  | 1345 | 1623 | 0.826 | 0.493-1.384 | 0.468 | R | | 11.610 | **0.009** | 74.160 | 0.77499 |
| ***GC* (rs4588)** | | | | | | | | | | | | |
| Allelic | 2 | 1078 | 1116 | 0.886 | 0.732-1.072 | 0.212 | F | | 0.553 | 0.457 | 0 | NA |
| Homozygous |  | 329 | 338 | 0.697 | 0.425-1.142 | 0.151 | F | | 0.088 | 0.767 | 0 | NA |
| Heterozygous |  | 509 | 515 | 0.942 | 0.735-1.208 | 0.64 | F | | 0.952 | 0.329 | 0 | NA |
| Dominant |  | 539 | 558 | 0.901 | 0.711-1.143 | 0.392 | F | | 0.850 | 0.357 | 0 | NA |
| Recessive |  | 539 | 558 | 0.708 | 0.437-1.147 | 0.160 | F | | 0.016 | 0.898 | 0 | NA |
| ***VDR* (ApaI: rs7975232)** | | | | | | | | | | | | |
| Allelic | 5 | 3660 | 4406 | 1.028 | 0.874-1.209 | 0.74 | R | | 11.306 | **0.023** | 64.622 | 0.891 |
| Homozygous |  | 947 | 1076 | 1.036 | 0.719-1.491 | 0.851 | R | | 13.455 | **0.009** | 70.272 | 0.999 |
| Heterozygous |  | 1350 | 1679 | 0.915 | 0.784-1.067 | 0.256 | F | | 2.043 | 0.728 | 0 | 0.191 |
| Dominant |  | 1830 | 2203 | 0.952 | 0.823-1.101 | 0.507 | F | | 5.053 | 0.282 | 20.846 | 0.435 |
| Recessive |  | 1830 | 2203 | 1.094 | 0.817-1.464 | 0.548 | R | | 12.579 | **0.014** | 68.202 | 0.771 |
| ^1^95% CI: 95% confidence interval; ^2^OR: odds ratio; ^3^R: random effects model; ^4^F: fixed effect model; Bold values indicate significance | | | | | | | | | | | | |


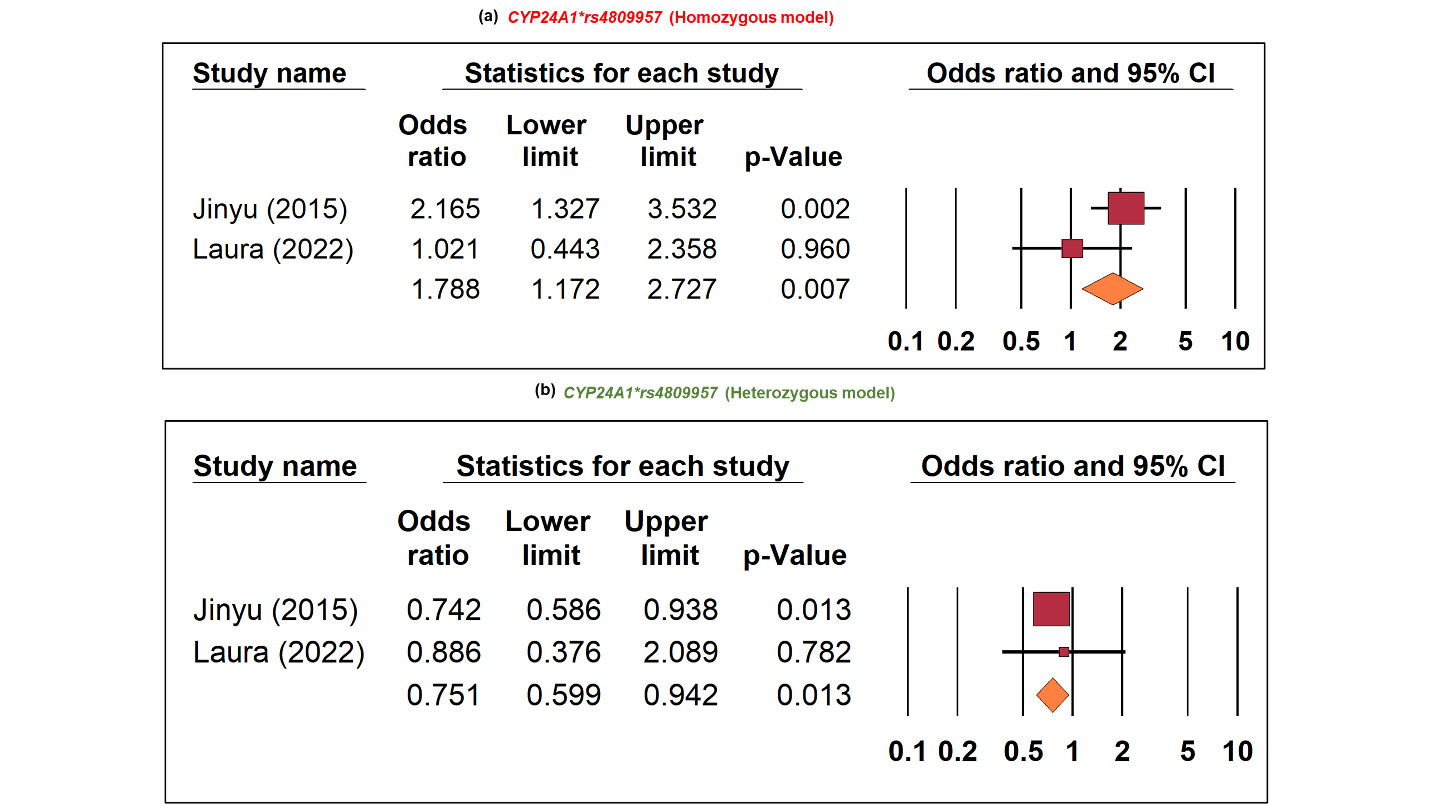


**Figure S1.** Forest plot of the pooled data for *CYP24A1*rs4809957* and increased lung cancer risk under (A) homozygous model while decreased under (B) heterozygous model; Red label indicates risk significance while green label indicates protective significance.


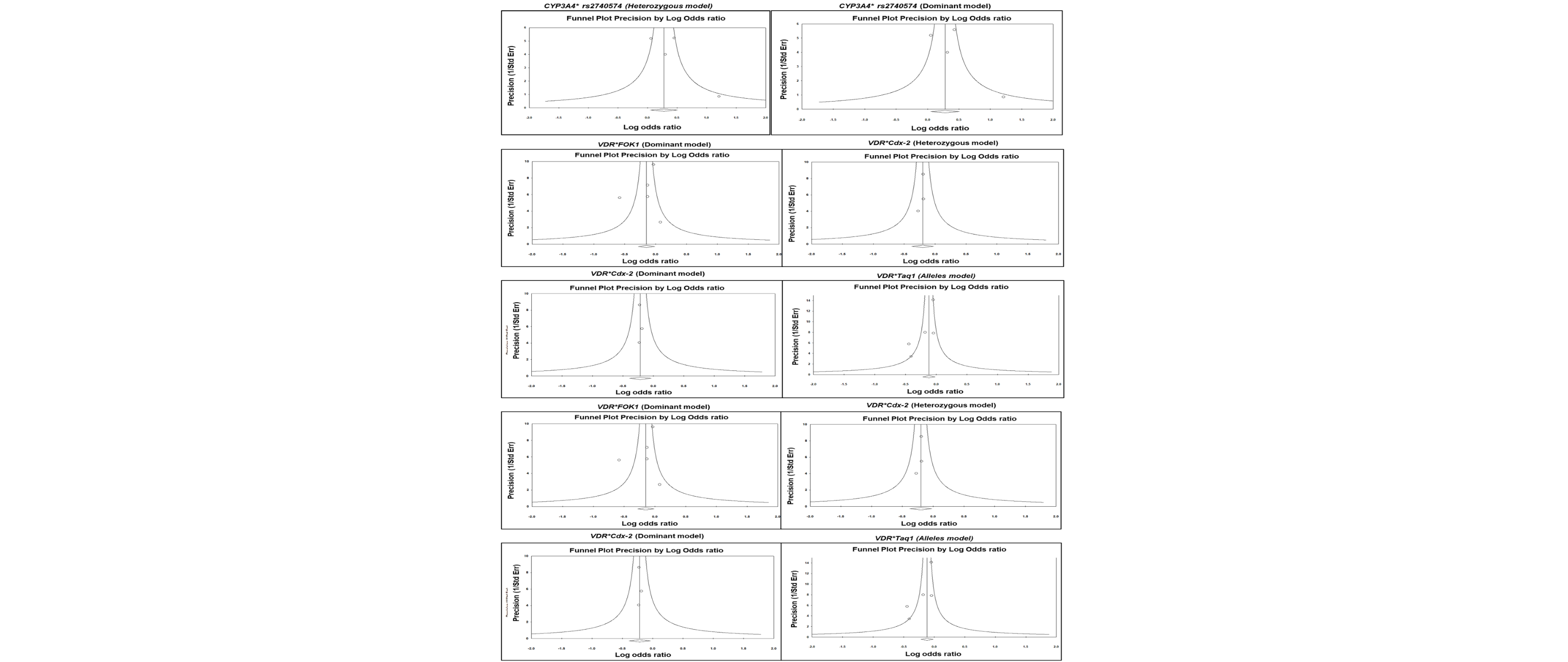


**Figure S2.** Funnel plot by Log odds ratio for selected variants showed symmetry indicating unavailability of publication bias.
